# Defining HIV Pre‐Exposure Prophylaxis (PrEP) Persistence: A Scoping Review

**DOI:** 10.1002/jia2.70115

**Published:** 2026-05-04

**Authors:** Sarah E. Rutstein, Grace E. Mulholland, Laura Limarzi‐Klyn, Annabelle Gallinek, Nicole Brown, William C. Miller

**Affiliations:** ^1^ School of Medicine University of North Carolina at Chapel Hill Chapel Hill North Carolina USA; ^2^ Gillings School of Global Public Health University of North Carolina at Chapel Hill Chapel Hill North Carolina USA

**Keywords:** adherence, engagement, HIV prevention, PrEP, prevention‐effective, surveillance

## Abstract

**Introduction:**

When assessing the effectiveness of pre‐exposure prophylaxis (PrEP) programmes, interventions, or modalities, it is important to understand patterns of PrEP use. Continued use of PrEP is frequently referred to as PrEP “persistence.” But persistence is not defined consistently, and differences impact the interpretation of study outcomes and public health policy. We conducted a scoping review to describe and compare definitions of PrEP persistence.

**Methods:**

We searched PubMed, Embase, Scopus and Global Health records (01/01/2012−01/26/2026) for results that discussed longitudinal anti‐HIV agents for HIV prevention. We included HIV, prevention and text variations of “persist‐.” We screened abstracts for relevance, reviewed relevant full‐text articles, and then extracted key outcomes. Screening and extraction were performed independently by two investigators; conflicts were reviewed and resolved by a third.

**Results:**

Our search returned 1549 de‐duplicated results. We reviewed 362 full‐text articles, yielding 147 studies for extraction. Approximately one‐third (42/147, 29%) provided only qualitative persistence definitions. Among studies with operational definitions (105/147; 71%), three‐quarters (80/105; 76%) considered a prescription refill and/or clinic visit date, and more than half (60/105; 57%) relied exclusively on these dates. Adherence (e.g. reported or measured drug taking) was commonly considered; 28% (29/105) of studies with an operational persistence definition included adherence assessment, and 11% (12/105) used only adherence to assess persistence. Thresholds used to classify persistent versus non‐persistent PrEP use varied considerably.

**Discussion:**

Definitions of PrEP persistence are heterogeneous. Most considered engagement in PrEP services (e.g. a clinic visit or medication refill), but nearly one‐third included or relied exclusively on adherence measures. The differences in definitions have important implications for cross‐study comparisons.

**Conclusions:**

The heterogeneity observed among persistence definitions complicates comparisons of PrEP interventions and related public health decision‐making. A single consensus definition of persistence is unlikely to suit all study settings, objectives, and designs; however, interpretability and comparability of results could be improved by increasing transparency and consistency in reporting. Our findings emphasize the importance of capturing clinically relevant, prevention‐effective use when possible and of rigorously considering the implications of a chosen persistence definition on estimates and associated conclusions.

## 1 Introduction

Biomedical HIV pre‐exposure prophylaxis (PrEP) is highly effective if protective levels are maintained during periods of HIV exposure [1, 2, 3, 4]. PrEP is a key pillar to end the global HIV epidemic, although PrEP use has not reached levels that are needed to achieve this goal [5, 6, 7, 8]. The Joint United Nations Programme on HIV/AIDS has previously set a goal of 21.2 million persons accessing PrEP globally by 2025 [9]. As of 2023, there were nearly 3.5 million persons using oral PrEP—a substantial and rapid increase over the prior 5 years, particularly in some of the most affected regions, including the African region, but far short of the global access goal. As PrEP modalities expand, including oral, long‐acting injectable, and removable vaginal rings, with even more options in the pipeline, the number of persons initiating PrEP is expected to increase [10, 11].

Characterizing the number of persons initiating, or at least prescribed, PrEP is an appealing metric in its ease of assessment, but the number of PrEP initiations alone does not reflect the potential impact of PrEP. Uptake among populations with the highest HIV incidence and indicators of ongoing use may be more relevant measures through which to appreciate the potential prevention afforded by increased PrEP access.

Sustained PrEP use throughout periods of HIV vulnerability is crucial to maximize the impact of PrEP on HIV prevention outcomes. Early discontinuation of PrEP (i.e. cessation of PrEP despite ongoing HIV vulnerability) is common and threatens the potential impact of PrEP [12, 13]. Continued PrEP use, often described as “PrEP persistence,” has emerged as a key concept to understanding the effectiveness (and cost‐effectiveness) of PrEP. Accordingly, persistence is a common outcome in PrEP‐related clinical and health services research.

Many interventions and implementation strategies seek to improve PrEP persistence through behavioural interventions, system modification to facilitate improved access, or even novel agents that extend the duration of coverage. But across these interventions and strategies, persistence is variably defined. In some contexts, the definition of persistence aligns more with drug adherence, a concept rooted in drug use and often measured by pill counts, patient self‐report or even concentrations of drug metabolite in biological specimens. Other definitions of persistence may refer to ongoing engagement in PrEP services, such as attendance at clinic visits or prescription refill, regardless of reported or measured adherence. And still other persistence definitions combine adherence and engagement measures. This heterogeneity can have profound implications: cross‐context comparisons of persistence become challenging, and the chosen persistence definition may affect programme evaluation outcomes, impacting whether a PrEP programme is considered successful and ultimately shaping public health policy and programmes.

Given the clinical and public health relevance of PrEP persistence, understanding the spectrum of persistence definitions can help researchers consider various approaches to operationalize this concept and aid in the interpretation of study outcomes. We conducted a scoping review to describe and compare definitions of HIV PrEP persistence in published literature, aiming to identify opportunities to improve coherence and consistency in characterizing PrEP persistence in future studies.

## 2 Methods

We developed our scoping review protocol using the Joanna Briggs Institute scoping review methodology informed by the Arksey and O'Malley Framework [14] and extensions by Levac et al. [15] and Peters et al. [16, 17]. The Arksey and O'Malley Framework outlines a multi‐stage process for conducting a scoping review: identifying the research question; identifying relevant studies; study selection, charting the data; and collating, summarizing and reporting the results. We also ensured alignment with the Preferred Reporting Items for Systematic reviews and Meta‐Analyses extension for Scoping Review (PRISMA‐ScR) checklist.

Two investigators (NB, LL‐K and GEM) independently searched PubMed, Embase, Scopus, and Global Health records for peer‐reviewed publications published between 1 January 2012 through 26 January 2026 for the following combination of terms: (1) text word variations on the word “persist”; (2) text words or controlled vocabulary terms for HIV; and (3) text words or controlled vocabulary terms for either pre‐exposure prophylaxis or for the combination of anti‐HIV agents and prevention. We did not apply any additional restrictions on the search, such as those related to language or publication status; however, only articles available in English were considered for full‐text review. The full electronic search strategy is provided in the Supplementary Appendix (Appendix A). The final search results were imported into Covidence (covidence.org), and duplicates were removed. The review of included articles was completed on 1 March 2026.

After compiling abstracts corresponding to the citations identified by the search, two investigators (from among NB, AG, GEM and LL‐K) independently screened each abstract. Conflicts were resolved by an additional investigator (SER). We retained any results that discussed longitudinal use of anti‐HIV agents for HIV prevention. We excluded results that did not fit the objective of the review, such as studies that focused solely on HIV treatment or were conducted specifically among people living with HIV. We also excluded papers that were not peer‐reviewed, such as medRxiv results. We did not require that the word “persistence” (or a variant) appear specifically in the abstract.

In the next stage, we attempted to identify full‐text articles for each retained result. We excluded conference abstracts. Two investigators (from among NB, GEM, AG, LL‐K or SER) independently screened the full‐text papers, with a final investigator (SER or GEM) resolving conflicts.

We did not require explicit definitions of persistence, so long as an implied definition was clear. For example, if the authors indicated in the Methods section that they estimated persistence, and the only longitudinal PrEP use results provided were estimates of the percent retained in PrEP services, we included the study and considered the authors’ definition of persistence to be retention in PrEP services. We excluded results where the definition of persistence was ambiguous (e.g. where authors indicated an intent to assess persistence but did not identify which of multiple results corresponded to persistence).

We developed a data charting form to capture, from each retained full‐text article, the study population, location/region of study, PrEP modality (or modalities) studied, study design, data source(s), persistence definition, and inputs for calculating persistence (if provided). We also identified whether each article characterized persistence “operationally” or “qualitatively,” with the former indicating a definition of persistence that uses specified data inputs (e.g. “returning for a PrEP follow‐up visit within 30 days”), and the latter indicating more conceptual definitions of persistence (e.g. “continued PrEP use”). Two investigators (from among NB, GEM, AG, LL‐K and SER) independently charted data from the eligible papers. Any conflicts in data characterization were reviewed and resolved (by SER or GEM).

### 2.1 Ethical Approval

Ethical approval was not required for this scoping review. The results of the scoping review consist exclusively of peer‐reviewed publications. As no human subjects were involved in the scoping review analysis of previously published work, no consent was obtained, nor was a waiver of consent required.

## 3 Results

**FIGURE 1:**
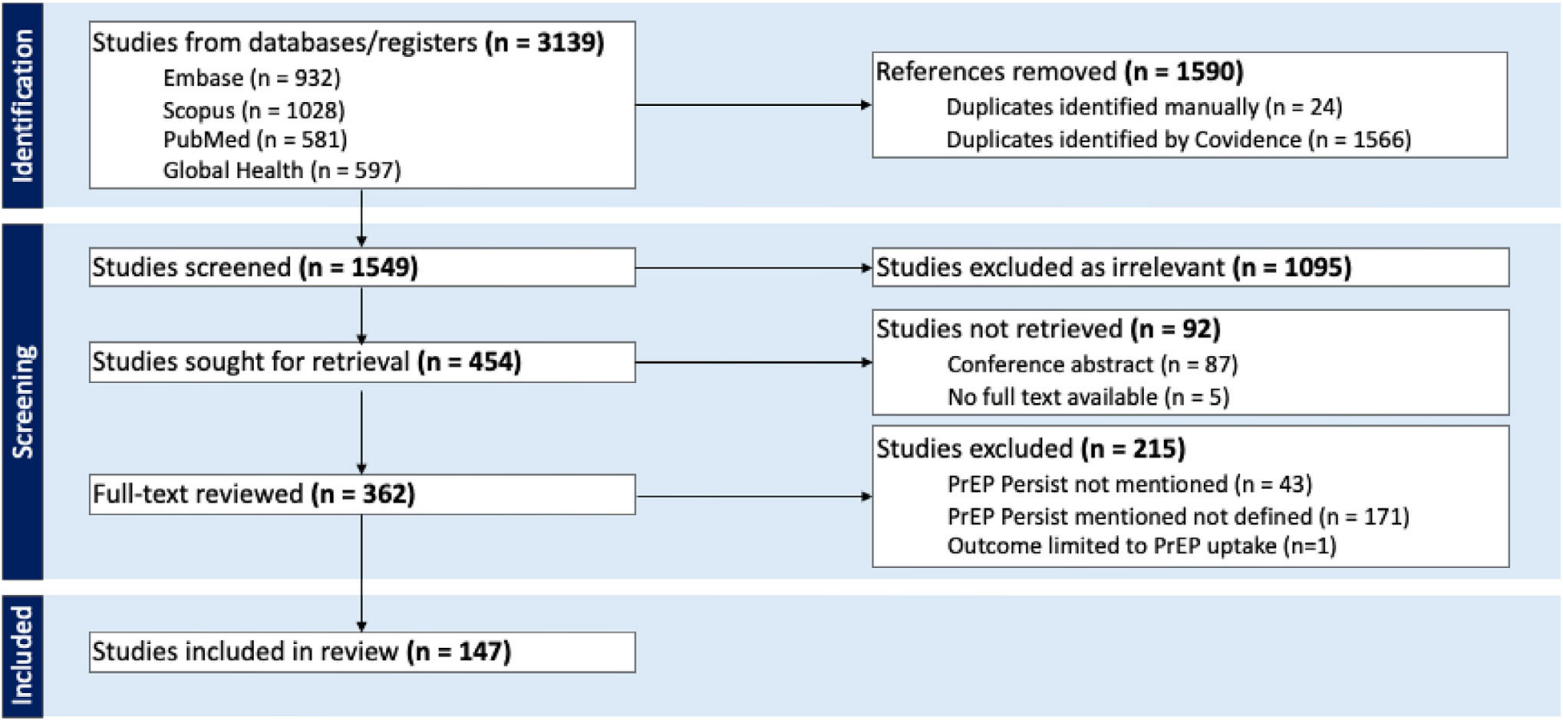
PRISMA diagram. Search results examining characterization of HIV PrEP persistence from PubMed, Embase, Scopus and Global Health records from 1 January 2012, through 26 January 2026.

Our initial search returned 3139 results; after duplicates were removed, 1549 remained (Figure 1). In the abstract screening process, we excluded 1095 results due to lack of relevance (e.g. study of treatment among persons living with HIV, no mention of HIV PrEP). Of the remaining 454 abstracts, 92 were excluded, primarily due to being published exclusively as a conference abstract (87/92), or without identified full‐text manuscripts corresponding to the identified abstract. In the full‐text screening phase, we excluded 215 results where the article mentioned PrEP persistence but did not define persistence (171/215; 79%) or the article did not mention PrEP persistence (43/215; 20%). Ultimately, we included 147 full‐text articles that defined PrEP persistence in our review (Table S1).

### 3.1 Study Characteristics

Most studies were conducted in North America (68/147; 46%), followed by Eastern or Southern Africa (58/147; 40%). The most common described population of the study was “all comers” (37/147; 25%), followed by men who have sex with men (29/147; 20%) and adolescent girls and young women (25/147; 17%). The most common study type was non‐experimental routine clinical data review, comprising 26% (38/147) of included studies. One‐quarter (37/147; 25%) of included studies were non‐experimental (e.g. observational) human subjects research. Ninety percent of studies were published since 2020 (133/147; 90%). Twelve of the studies explicitly evaluated PrEP persistence among long‐acting injectable agents [18, 19, 20, 21, 22, 23, 24, 25, 26, 27, 28, 29], and four studies included evaluation of persistence with the vaginal ring [21, 30, 31, 32].

### 3.2 Defining Persistence

Among the included 147 studies, 42 (29%) provided only a qualitative definition of PrEP persistence, identifying relevant concepts (e.g. “continued on PrEP”) without providing specific inputs that would facilitate operationalizing the definition in the context of a programme evaluation or clinical records review. The remaining studies (105/147; 71%) included a definition of persistence deemed as “operational,” in which a threshold and input or inputs were provided through which persistence could be assessed and applied, internally and externally. Just under half (47/105; 44%) of studies with operational persistence definitions were conducted in North America, with the remaining in Eastern or Southern Africa (48/105; 45%), Europe (4/105; 4%) or Asia (5/105; 5%). The most common study design was a non‐experimental routine clinical data review (35/105; 33%), followed by experimental human subjects research (29/105; 27%), and then non‐experimental (observational) human subjects research (21/105; 20%).

**FIGURE 2:**
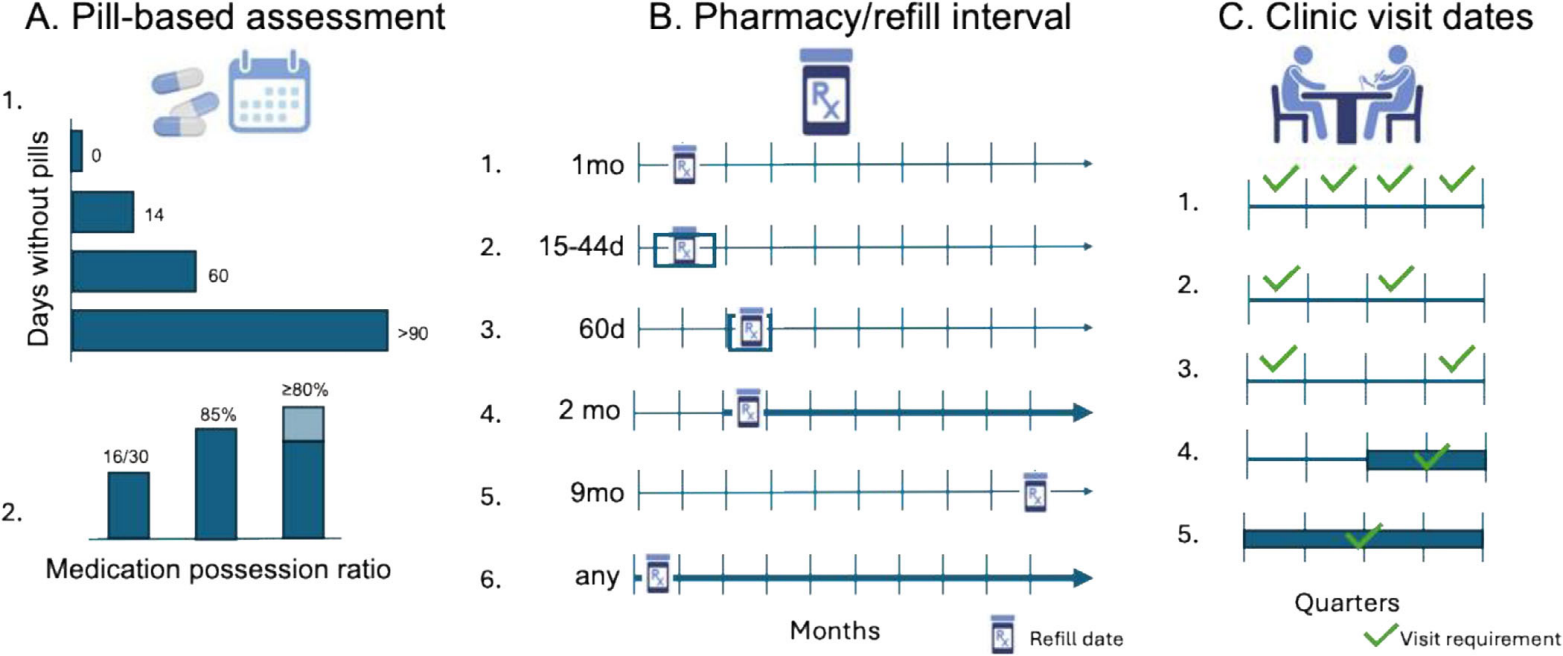
HIV PrEP persistence definitions. We observed substantial heterogeneity across HIV PrEP persistence inputs among the 105 studies with operational definitions of PrEP persistence, as depicted by a sample of definitions represented in the above figure. Definitions relied on a mix of pill‐based, pharmacy/refill and clinic visit date increments and cut‐offs through which persistence outcomes were characterized or defined. Examples of how persistence was defined are further described below: A. 1. a pill‐based assessment calculating the number of days a person may be without pills, with various thresholds (0, 14, 60 or >90 days) as consistent with non‐persistence. A. 2. Medication possession ratio, in which percent of days covered characterizes whether a person is persistent. B. 1. A refill 1 month following initiation. B. 2. A refill occurring anywhere between 15 and 44 days after initiation. B. 3. A single refill 60 days following initiation. B. 4. A refill occurring anytime after 2 months after initiation. B. 5. A refill occurring 9 months after initiation. B. 6. Any refill, at any point, following initiation. C. 1. At least one clinic visit in each quarter for the four quarters (1 year) following initiation. C. 2. One visit in the first and third quarter of the year following initiation. C. 3. Two visits, at least 6 months apart, in the year following initiation. C. 4. Any visit in the 6–12 months following initiation. C. 5. Any visit at any time in the 12 months following initiation.

Conceptually, persistence definitions generally fell into three categories: pill‐based assessments, which included both objectively measured and self‐reported adherence; pharmacy refill dates; and indicators of care engagement based on clinic visits. Tremendous heterogeneity was observed across these categories (Figure 2). For example, one study may define PrEP persistence as having sufficient pills to cover 80% of daily dosing, another may define persistence as receiving any refill after PrEP initiation, and another may define persistence based on on‐time attendance at quarterly clinic visits.

**FIGURE 3:**
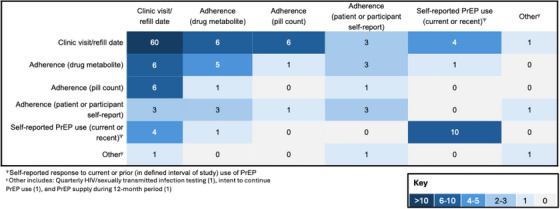
Relative frequency and combination of defined persistence inputs. Examining the frequency and concurrent inputs used to define HIV PrEP persistence among 147 studies that included an operational definition of persistence. Clinic visit date or PrEP prescription refill date was the most frequently included input, often as the only parameter used to characterize PrEP persistence. PrEP use, as distinct from PrEP adherence, was also common, often captured with survey questions evaluating self‐reported “current” or “continued” use at time of assessment.

Among studies with operational persistence definitions, three‐quarters (80/105; 76%) assessed persistence using prescription refill and/or clinic visit dates, with more than half (60/105; 57%) relying exclusively on refill and/or clinic visit dates to characterize persistence (Table S2 and Figure 3). Adherence was also commonly used as an input for assessing persistence. Measures of adherence varied and included measures of blood metabolite levels, pill counts and self‐reported PrEP utilization. Approximately one quarter of studies (29/105; 28%) included some assessment of adherence, with 11% (12/105) including only measures of adherence in their characterization of PrEP persistence. Finally, 14% of studies (15/105) relied on patient self‐report of current or recent PrEP use, with 9% (10/105) using this metric as the only input to assess PrEP persistence.

Objective markers of adherence, specifically quantitative metabolite‐based assays, were more commonly applied in experimental human subjects’ studies (10/29; 34%) than in non‐experimental human subjects’ studies (3/56; 5%). Non‐experimental, routine clinical data reviews largely relied on clinic visit or refill dates to assess persistence.

## 4 Discussion

Unlike with HIV treatment adherence, where viral suppression can serve as objective evidence of adequate exposure, the fluctuating nature of HIV exposure risk presents a challenge for defining an outcome that most appropriately captures effective PrEP use [33]. Persistence has emerged as a term that generally seeks to capture the concept of continued use. Persistence has been increasingly identified and defined in PrEP studies—from fewer than five studies per year in 2012–2019, to more than 20 per year since 2022.

Among 147 studies that included any definition of PrEP persistence, persistence definitions were heterogeneous. The most common input used to assess persistence was the date of a clinic visit or prescription refill. Visit or refill dates were used in more than three‐quarters of studies that provided an operational definition of persistence; it was the only input for persistence for more than half. This frequent reliance on visit or refill dates across studies likely reflects data availability but also suggests that continued engagement in PrEP services is perceived as a key component of PrEP persistence. The inclusion of adherence assessments as part of a persistence definition was less common, observed in only a quarter of studies with an operational definition of PrEP persistence.

The relevance of various inputs for assessing ongoing, prevention‐effective PrEP use will likely evolve with the expansion of additional PrEP modalities. The vast majority of studies included in this scoping review evaluated oral PrEP, the PrEP modality most widely studied and available to date. Other PrEP modalities, such as the vaginal ring or long‐acting injectable agents, were the focus of only a few included studies [18, 19, 20, 21, 22, 23, 24, 25, 26, 27, 28, 29, 30, 31, 32]. Long‐acting, clinic‐administered agents may ultimately eliminate considerations of adherence in assessments of ongoing PrEP coverage. For such agents, engagement in PrEP services—that is receiving an injection at a clinic visit—confers protection for a pre‐determined period. However, if these agents evolve to home‐based self‐administration, indicators of adherence will again be pertinent.

Furthermore, inputs used to assess persistence in the context of daily oral PrEP may not be optimal for assessing persistence in the context of event‐driven PrEP. For example, studies of daily oral PrEP that considered adherence as a persistence input could be guided by established adherence thresholds for effective use of daily oral PrEP. Characterizing adherence in the context of event‐driven PrEP would be more challenging given the expectation of fluctuating PrEP needs. For event‐driven PrEP, PrEP non‐adherence may align with periods without HIV vulnerability rather than lapses in HIV protection. In this case, an engagement‐related input, such as timely refills, would likely be more useful for defining PrEP persistence than adherence measures that do not consider fluctuations in HIV vulnerability. We did not distinguish between daily oral and event‐driven PrEP in this scoping review. However, two studies explicitly defined persistence in the context of event‐driven PrEP: one exclusively considered engagement (i.e. on‐time—within 14‐day—attendance at quarterly clinic visits) [27], and the second considered adherence (i.e. self‐reported adherence to event‐driven dosing regimen in the prior 30 days, with persistence defined based on sex acts and corresponding tenofovir diphosphate (TFVdp) concentrations felt appropriate for that potential exposure) [29].

As shorter‐interval (e.g. daily oral or event‐driven oral PrEP) are expected to remain in the PrEP landscape, and the potential for out‐of‐clinic administration of long‐acting agents (self‐injections, patches, monthly pills, etc.) expands, our review highlights the importance of specifying the potential impact of persistence definition inputs. Specifically, an increased attention to and reporting of the implications of alternative definitions with varying thresholds of adherence‐ or engagement‐based persistence cutoffs, and the expected performance or accuracy as assessed (e.g. the precision of metabolite‐based assessment vs. self‐report).

The findings of this review expose limitations in comparisons of persistence estimates across studies and settings. We primarily focused on the diversity of inputs, yet even among papers with similar persistence inputs, we found substantial heterogeneity in the thresholds used to categorize PrEP use as persistent. For example, among articles that used prescription refill data to assess persistence, persistence definitions included: no lapse in PrEP coverage longer than 14 days [34], 30 days [35] or 90 days [36]; no gaps in pill coverage at all [37]; attendance at quarterly refill visits [38]; at least 16 days of pill coverage per 30 days for at least three‐quarters of a period [39]; returning for any PrEP refills [40], at least one refill in a 3‐month period [41]; and so on. This additional variation in PrEP definitions further complicates efforts to compare persistence across populations and the effectiveness of tested interventions.

To our knowledge, this review is the first to consolidate published definitions of persistence using a rigorous process to identify, review and extract data from relevant studies. Despite our systematic approach, we may have underestimated the true diversity in definitions, as our results may include multiple publications from the same data source or research groups, inheriting the same definition of persistence from the parent study. Excluding papers that were not published in English could also limit generalizability. Our extraction approach focused on PrEP modality (injectable, oral, vaginal ring) but did not necessarily distinguish between PrEP dosing (daily oral vs. event‐driven/on demand), which limits our ability to compare variation in persistence definitions between these prescription strategies. Finally, we did not extract whether a prescription was provided from an on‐line service provider or in‐person, the former emerging as an increasingly popular means of PrEP prescriptions. Although this omission does not bias the articles reviewed, we are not able to comment on how or if there are differences in PrEP persistence inputs using on‐line PrEP provision, though we would expect these platforms to rely more on prescription fill dates based on the remote nature of care provision.

We do not posit that a single definition of persistence is superior. While the selection of inputs for persistence definition may be guided by a conceptualization of relevant factors, we acknowledge that it is likely constrained by available data and resources. For example, it may not be practical to use dates of clinic visits or refills if patients engage in PrEP services at numerous facilities, if the relevant facilities do not have the capacity to facilitate access to the records, or if the relevant records are paper‐based. Additionally, metabolite‐based assessments of PrEP adherence are not feasible in larger‐scale observational studies or retrospective programme evaluations. How or if less expensive biomarkers of adherence, such as urine‐based metabolite testing, could penetrate clinical practice is not known [42, 43]. The relative impact of these assays in characterizing PrEP persistence should be explored in comparison to other adherence assessments, including blood‐based metabolites and visit dates with pill possession ratios.

In our review, only three studies accounted for a person stopping PrEP after being determined “low risk” in consultation with a healthcare provider [27, 29, 44], and two studies offered an alternative persistence threshold for persistence in the context of participants reporting sexual activity [29, 45]. This finding suggests that most persistence definitions do not characterize *prevention‐effective* persistence, a paradigm that captures the dynamics of HIV risk and PrEP coverage. Specifically, prevention‐effective use describes the relevance of PrEP coverage insomuch as that coverage occurs during periods of HIV vulnerability [33]. We anticipate the dearth of outcomes that consider prevention‐effective persistence is due to the challenges obtaining reliable behavioural data that can be cross‐referenced with biomarker data—sex diaries, timeline follow‐backs, and even two‐way messaging can all offer granular insights into HIV risk, but come at a substantial cost, and are not easily triangulated to periods of PrEP protection for shorter interval (i.e. not long‐acting) PrEP agents. Stopping or pausing PrEP may be clinically appropriate if related to lack of indication [33, 46, 47], and failure to consider the role of fluctuating PrEP indications in characterizing PrEP persistence suggests an important gap in some of the most clinically relevant endpoints related to PrEP's role in HIV prevention. Increased attention to prevention‐effective use as part of persistence definitions can help move towards a more clinically relevant characterization of PrEP use outcomes.

Unlike for persons living with HIV, in which the appropriate clinical and public health objective is lifelong viral suppression and where targets can help guide resource allocation and programme implementation [48], the objective for PrEP as part of a comprehensive HIV prevention strategy is more complex. HIV prevention cascades have been proposed, and are appealing conceptually but challenging to implement given the dynamic nature of PrEP need [49]. Adequate and appropriate coverage of PrEP during periods of HIV vulnerability will be crucial to maximize PrEP's prevention potential, and persistence will undoubtedly continue to take a central role in PrEP outcomes. In the absence of a standardized definition of persistence, researchers should examine the implications of various inputs and thresholds by conducting sensitivity analyses within available data. Such efforts can help to improve our understanding of the extent of variability in persistence estimates that is attributable to differences in how persistence is defined. These analyses could inform guidelines to improve consistency across reports of PrEP persistence and other metrics of longitudinal PrEP use.

## 5 Conclusions

Our findings highlight the diversity among definitions of PrEP persistence and suggest that these differences influence conclusions related to PrEP effectiveness across implementation, programme evaluation and intervention studies, obscuring public health decision‐making that seeks to maximize HIV prevention through PrEP. A single consensus definition of persistence is unlikely to suit all study settings, objectives and designs; however, interpretability and comparability of results could be improved by increasing transparency and consistency in reporting. Our findings emphasize the importance of using available data to capture clinically relevant prevention‐effective use when able, and rigorously considering the implications of a chosen persistence definition on estimates and associated conclusions. Besides examining the implications within a single study, future work should examine the relationship between alternative persistence components (engagement, adherence and the mix therein), and the observed impact on HIV prevention efforts at a population level.

## Author Contributions

SER conceptualized manuscript content and objectives. NB, LL‐K, GEM, AG and SER reviewed abstracts, full‐text articles and contributed to data extraction or adjudication (SER and GEM). All authors contributed to manuscript drafts and revisions, and all have reviewed and approved the version as submitted.

## Funding

GEM was supported by the National Institute of Allergy and Infectious Diseases of the National Institutes of Health under award number T32‐AI007001. SER received support from the National Institue of Allergy and Infectious Diseases of the National Institutes of Health under award R61‐AI174285.

## Conflicts of Interest

The authors declare no conflicts of interest.

## Supporting information




**Supporting Information File 1**: Supplemental Tables 1 and 2.
**Supporting Information File 2**: Appendix A—search strategy.

## Data Availability

All data pertaining to full‐text articles are available in Table S1. Additional data capturing all searched abstracts are available on request.
