# Supplementary material for: Defining HIV Pre‐Exposure Prophylaxis (PrEP) Persistence: A Scoping Review
Source: J Int AIDS Soc. 2026 May 4;29(5):e70115. doi: 10.1002/jia2.70115 (PMC13139717; doi:10.1002/jia2.70115)
Supplement: Supplementary file 1 — Supporting Information File 1: Supplemental Tables 1 and 2. Supporting Information File 2: Appendix A—search strategy. [file JIA2-29-e70115-s001.docx]

# Appendix A

The full electronic search strategies are provided in the table below.

| **Database** | **Search Strategy** | **Limitations** | **Results** |
| --- | --- | --- | --- |
| Embase | (('pre-exposure prophylaxis'/exp OR  (prophylaxis:ti,ab AND (pre-exposure:ti,ab OR 'pre exposure':ti,ab OR pre-exposure:kw OR 'pre exposure':kw)) OR  (prophylaxis:kw AND (pre-exposure:ti,ab OR 'pre exposure':ti,ab OR pre-exposure:kw OR 'pre exposure':kw))) OR  ((('primary prevention'/exp OR  prevent*:ti,ab OR prevent*:kw) AND (HIV/exp OR  'human immunodeficiency virus':ti,ab OR 'human immunodeficiency virus':kw OR  HIV:ti,ab OR HIV:kw)) AND ('anti-HIV agent'/exp OR  'anti human immunodeficiency virus agent':ti,ab OR 'anti human immunodeficiency virus agent':kw OR  'anti-human immunodeficiency virus agent':ti,ab OR 'anti-human immunodeficiency virus agent':kw OR  'anti HIV agent':ti,ab OR 'anti HIV agent':kw OR  'anti-HIV agent':ti,ab OR 'anti-HIV agent':kw))) AND  (persist*:ti,ab OR persist*:kw) | Year >= 2012 | 882 |
| Global Health | ((DE (pre-exposure prophylaxis) OR  TI (prophylaxis AND TI (pre-exposure OR pre exposure)) OR  AB (prophylaxis AND (pre-exposure OR pre exposure)) OR  ID (prophylaxis AND (pre-exposure OR pre exposure)) OR  (((DE (disease prevention) OR  TI (prevent*) OR AB (prevent*) OR ID (prevent*)) AND (DE (human immunodeficiency viruses) OR  TI (human immunodeficiency virus) OR AB (human immunodeficiency virus) OR ID (human immunodeficiency virus) OR  TI (HIV) OR AB (HIV) OR ID (HIV))) AND (DE (antiretroviral agents) OR  TI (anti human immunodeficiency virus agent) OR AB (anti human immunodeficiency virus agent) OR ID (anti human immunodeficiency virus agent) OR  TI (anti-human immunodeficiency virus agent) OR AB (anti-human immunodeficiency virus agent) OR ID (anti-human immunodeficiency virus agent) OR  TI (anti HIV agent) OR AB (anti HIV agent) OR ID (anti HIV agent) OR  TI (anti-HIV agent) OR AB (anti-HIV agent) OR ID (anti-HIV agent)))) AND  (TI (persist*) OR AB (persist*) OR ID (persist*)) | Year >= 2012 | 425 |
| PubMed | ( TITLE-ABS-KEY ( pre-exposure prophylaxis ) OR ( TITLE-ABS-KEY ( prophylaxis ) AND ( TITLE-ABS-KEY ( pre-exposure ) OR ( TITLE-ABS-KEY ( pre exposure ))))) OR  (( TITLE-ABS-KEY ( "primary prevention" ) OR (TITLE-ABS-KEY ( prevent* ) AND ( TITLE-ABS-KEY ( hiv ) OR TITLE-ABS-KEY ( "human immunodeficiency virus" ))) AND ( TITLE-ABS-KEY ( "anti-HIV agent" ) OR TITLE-ABS-KEY ( "anti human immunodeficiency virus agent" ) OR TITLE-ABS-KEY ( "anti-human immunodeficiency virus agent" ) OR TITLE-ABS-KEY ( "anti HIV agent" ) OR TITLE-ABS-KEY ( "anti-HIV agent" ) ) ) ) AND  ( TITLE-ABS-KEY ( persist* )) | Year >= 2012 | 622 |

**Supplemental Table 1.** Studies with PrEP persistence defined

| **Author** | **Publication year** | **Region** | **Study population** | **Study design** | **PrEP modality** | **Persistence definition (Operational or Qualitative)** |
| --- | --- | --- | --- | --- | --- | --- |
| Abuogi, L^50^ | 2025 | North America | Other: Youth 14-24 | Experimental - human subjects | oral PrEP | Operationally |
| Amico, K.^51^ | 2017 | Eastern/Southern Africa | Women (all ages) | Qualitative research | oral PrEP | Qualitatively |
| Amico, K^52^ | 2014 | Not specified | All comers | Other: commentary | not specified | Qualitatively |
| Argenyi, M^53^ | 2022 | North America | All comers | Non-experimental - Routine clinical data review | Presumed oral PrEP | Operationally |
| Arnold, T^54^ | 2025 | North America | MSM | Other: Protocol paper | Presumed oral PrEP | Operationally |
| Baeten, J^30^ | 2021 | Eastern/Southern Africa | Women (all ages) | Experimental - human subjects | vaginal ring | Operationally |
| Barnabee, G^55^ | 2022 | Eastern/Southern Africa | AGYW | Experimental - human subjects | oral PrEP | Operationally |
| Barnabee, G^56^ | 2023 | Eastern/Southern Africa | AGYW | Non-experimental - Routine clinical data review | oral PrEP | Operationally |
| Bassett, I^57^ | 2026 | Eastern/Southern Africa | AGYW | Experimental - human subjects | oral PrEP | Operationally |
| Bazzi, A^18^ | 2023 | North America | PWID | Experimental - human subjects | oral PrEP; injectable PrEP | Operationally |
| Bien-Gund, C^58^ | 2022 | Eastern/Southern Africa | Women (all ages) | Experimental - human subjects | Presumed oral PrEP | Operationally |
| Blumenthal, J^59^ | 2021 | North America | Women (all ages) | Experimental - human subjects | oral PrEP | Operationally |
| Bogart, L^60^ | 2024 | Eastern/Southern Africa | All comers | Experimental - human subjects | oral PrEP | Operationally |
| Bondarchuk, C^25^ | 2026 | Eastern/Southern Africa | Men (all ages) | Review article | oral PrEP; injectable PrEP | Qualitatively |
| Braun, R^61^ | 2023 | North America | MSM; Transgender persons | Experimental - human subjects | Presumed oral PrEP | Operationally |
| Bruxvoort, K^62^ | 2021 | North America | All comers | Non-experimental - Routine clinical data review | Presumed oral PrEP | Operationally |
| Bui, H^27^ | 2024 | Asia | MSM | Other: protocol paper | oral PrEP; injectable PrEP | Operationally |
| Butts, S^63^ | 2025 | North America | MSM | Experimental - human subjects | Presumed oral PrEP | Qualitatively |
| Celum, C^64^ | 2019 | Eastern/Southern Africa | AGYW | Other: Commentary | oral PrEP | Qualitatively |
| Celum, C^65^ | 2020 | Eastern/Southern Africa | AGYW | Experimental - human subjects | oral PrEP | Operationally |
| Celum, C^66^ | 2021 | Eastern/Southern Africa | AGYW | Experimental - human subjects | oral PrEP | Operationally |
| Celum, C^67^ | 2022 | Eastern/Southern Africa | AGYW | Non-experimental - human subjects | oral PrEP | Operationally |
| Celum, C^68^ | 2023 | Eastern/Southern Africa | AGYW | Experimental - human subjects | Presumed oral PrEP | Operationally |
| Chan, P^69^ | 2025 | North America | MSM | Non-experimental - human subjects | Presumed oral PrEP | Operationally |
| Chapin-Bardales, J^70^ | 2023 | North America | MSM | Non-experimental - human subjects | oral PrEP | Operationally |
| Chase, E^71^ | 2022 | North America | All comers | Non-experimental - Routine clinical data review | Presumed oral PrEP | Operationally |
| Chen, Y^72^ | 2024 | North America | MSM | Non-experimental - Routine clinical data review | oral PrEP | Operationally |
| Chen-Charles, J^73^ | 2025 | Eastern/Southern Africa | AGYW | Review article | oral PrEP | Qualitatively |
| Chipukuma, J^74^ | 2023 | Eastern/Southern Africa | AGYW | Non-experimental - Routine clinical data review | oral PrEP | Operationally |
| Clement, M^75^ | 2019 | North America | All comers | Non-experimental - Routine clinical data review | Presumed oral PrEP | Operationally |
| Clement, M^76^ | 2021 | North America | All comers | Non-experimental - Routine clinical data review | Presumed oral PrEP | Operationally |
| Colver, J^77^ | 2025 | North America | Other: active-duty military | Non-experimental - Routine clinical data review | oral PrEP | Operationally |
| Coy, K^39^ | 2019 | North America | All comers | Non-experimental - Routine clinical data review | oral PrEP | Operationally |
| Davey, D^45^ | 2022 | Eastern/Southern Africa | Pregnant or breastfeeding women | Other: protocol paper | oral PrEP | Operationally |
| Davey, D^78^ | 2025 | Eastern/Southern Africa | AGYW; Men (all ages); Other: young people | Experimental - human subjects | oral PrEP | Operationally |
| de Voux, A^79^ | 2023 | Eastern/Southern Africa | Pregnant or breastfeeding women | Experimental - human subjects | oral PrEP | Operationally |
| Dean, L^80^ | 2021 | North America | All comers | Non-experimental - Routine clinical data review | Presumed oral PrEP | Operationally |
| Doblecki-Lewis, S^81^ | 2024 | North America | All comers | Non-experimental - Routine clinical data review | Presumed oral PrEP | Operationally |
| Evans, K^82^ | 2022 | North America | Other: Black and Hispanic/Latino persons | Review article | not specified | Qualitatively |
| Fan, C^83^ | 2025 | Asia | MSM | Experimental - human subjects | oral PrEP | Operationally |
| Felker-Kantor, E^84^ | 2023 | Eastern/Southern Africa | Men (all ages) | Non-experimental - Routine clinical data review | oral PrEP | Operationally |
| Fischer, A^85^ | 2025 | Eastern/Southern Africa | AGYW | Review article | not specified | Operationally |
| Furukawa, N^86^ | 2020 | North America | All comers | Non-experimental - human subjects | Presumed oral PrEP | Qualitatively |
| Garrison, L^46^ | 2021 | North America | Not specified | Review article | Presumed oral PrEP | Qualitatively |
| Gichane, M^87^ | 2025 | Eastern/Southern Africa | AGYW | Experimental - human subjects | oral PrEP | Operationally |
| Gillespie, D^88^ | 2022 | Europe | MSM | Non-experimental - human subjects | Presumed oral PrEP | Qualitatively |
| Goedel, W^89^ | 2022 | North America | MSM | Non-experimental - human subjects | Presumed oral PrEP | Qualitatively |
| Goedel, W^90^ | 2022 | North America | MSM | Experimental - human subjects | oral PrEP | Operationally |
| Haberer, J^91^ | 2023 | Other: Global | Not specified | Review article | oral PrEP | Qualitatively |
| Hall, C^92^ | 2022 | North America | MSM | Qualitative research | oral PrEP | Qualitatively |
| Haribhai, S^93^ | 2023 | Eastern/Southern Africa | Other: pregnant AGYW | Non-experimental - human subjects | oral PrEP | Operationally |
| Heffron, R^94^ | 2025 | Eastern/Southern Africa | Women (all ages) | Experimental - human subjects | oral PrEP | Operationally |
| Hendrickson, C^44^ | 2020 | Eastern/Southern Africa | Not specified | Review article | oral PrEP | Operationally |
| Hendrickson, C^95^ | 2025 | Eastern/Southern Africa | MSM; FSW/sex worker; AGYW | Other: cost-effectiveness | oral PrEP | Operationally |
| Hill, N^96^ | 2025 | North America | Other: Youth aged 18-25 | Non-experimental - Routine clinical data review | Presumed oral PrEP | Operationally |
| Hodges-Mameletzis, I^97^ | 2019 | Not specified | Women (all ages) | Review article | Other: No specific focus, discussed studies of various modalities | Qualitatively |
| Holloway, I^98^ | 2020 | North America | MSM | Non-experimental - human subjects | oral PrEP | Operationally |
| Huang, YL^35^ | 2021 | North America | All comers | Non-experimental - Routine clinical data review | Presumed oral PrEP | Operationally |
| Isehunwa, O^99^ | 2024 | Eastern/Southern Africa | Pregnant or breastfeeding women; Women (all ages) | Non-experimental - human subjects | oral PrEP | Operationally |
| Jackson-Gibson, M^100^ | 2021 | Eastern/Southern Africa | AGYW | Non-experimental - human subjects | Presumed oral PrEP | Qualitatively |
| Jenness, S^101^ | 2021 | North America | MSM | Other: Model | Presumed oral PrEP | Qualitatively |
| Jiang, H^102^ | 2025 | Asia | MSM | Other: Other: "Viewpoint" | not specified | Qualitatively |
| Jin, E^24^ | 2025 | Eastern/Southern Africa | AGYW | Other: Modeling study | oral PrEP; injectable PrEP | Operationally |
| John, S^103^ | 2017 | North America | MSM | Non-experimental - human subjects | Presumed oral PrEP | Qualitatively |
| John, S^104^ | 2019 | North America | MSM | Non-experimental - human subjects | Presumed oral PrEP | Operationally |
| Jones, J^105^ | 2022 | North America | MSM | Non-experimental - human subjects | oral PrEP | Operationally |
| Jones, J^106^ | 2022 | North America | MSM | Non-experimental - human subjects | oral PrEP | Qualitatively |
| Keyes, J^107^ | 2020 | North America | All comers | Non-experimental - Routine clinical data review | oral PrEP | Operationally |
| Khadka, N^108^ | 2023 | Eastern/Southern Africa | Other: Pregnant and postpartum AGYW | Non-experimental - human subjects | oral PrEP | Operationally |
| Khati, A^109^ | 2025 | North America | PWID | Experimental - human subjects | oral PrEP | Operationally |
| Khosropour, C^110^ | 2023 | North America | Other: MSM, transgender women, or cisgender women with specific risk factors for HIV or other STIs | Non-experimental - human subjects | Presumed oral PrEP | Qualitatively |
| Kumar, R^28^ | 2024 | Eastern/Southern Africa | FSW/sex worker | Other: Protocol paper | oral PrEP; injectable PrEP | Operationally |
| Kusemererwa, S^20^ | 2025 | Eastern/Southern Africa | Men (all ages) | Experimental - human subjects | oral PrEP; injectable PrEP | Operationally |
| Laborde, N^111^ | 2020 | North America | All comers | Non-experimental - human subjects | oral PrEP | Qualitatively |
| Lagat, H^32^ | 2024 | Eastern/Southern Africa | AGYW | Experimental - human subjects | oral PrEP; vaginal ring | Operationally |
| Le Roux, C^112^ | 2024 | Europe | All comers | Non-experimental - Routine clinical data review | oral PrEP | Operationally |
| Lelutiu-Weinberger, C^113^ | 2024 | Europe | MSM | Other: Protocol paper | oral PrEP | Qualitatively |
| Matsuno, A^114^ | 2025 | Eastern/Southern Africa | AGYW | Non-experimental - human subjects | not specified | Operationally |
| Matthews, L^115^ | 2024 | Eastern/Southern Africa | Women (all ages) | Non-experimental - human subjects | oral PrEP | Operationally |
| McCormick, C^116^ | 2023 | North America | All comers | Non-experimental - Routine clinical data review | oral PrEP | Operationally |
| McNulty, M^117^ | 2023 | North America | Other: PrEP-providing organizations representatives | Non-experimental - human subjects | not specified | Qualitatively |
| Mirembe, B^118^ | 2024 | Eastern/Southern Africa | AGYW | Experimental - human subjects | oral PrEP | Operationally |
| Moon, E^119^ | 2025 | Europe | All comers | Non-experimental - Routine clinical data review | oral PrEP | Operationally |
| Mulholland, G^120^ | 2025 | Eastern/Southern Africa | All comers | Non-experimental - Routine clinical data review | oral PrEP | Operationally |
| Naz-McLean, S^121^ | 2024 | Central/South America | Transgender persons | Non-experimental - human subjects | Presumed oral PrEP | Qualitatively |
| Ndimande-Khoza, M^122^ | 2023 | Eastern/Southern Africa | AGYW | Non-experimental - human subjects | Presumed oral PrEP | Qualitatively |
| Oglesby, A^123^ | 2024 | North America | All comers | Non-experimental - Routine clinical data review | oral PrEP | Operationally |
| Ohiomoba, R^124^ | 2022 | Eastern/Southern Africa | AGYW | Non-experimental - human subjects | oral PrEP | Qualitatively |
| Onwubiko, U^125^ | 2024 | North America | MSM | Non-experimental - human subjects | oral PrEP | Operationally |
| O’Rourke,S^126^ | 2021 | Eastern/Southern Africa | AGYW | Experimental - human subjects | oral PrEP | Operationally |
| Pike, C^21^ | 2025 | Eastern/Southern Africa | Other: young people 15-29 | Other: Protocol paper | oral PrEP; injectable PrEP; vaginal ring | Operationally |
| Pintye, J^127^ | 2021 | Eastern/Southern Africa | AGYW | Non-experimental - human subjects | oral PrEP | Qualitatively |
| Pintye, J^128^ | 2023 | Eastern/Southern Africa | Pregnant or breastfeeding women | Experimental - human subjects | oral PrEP | Operationally |
| Platt, L^129^ | 2023 | North America | All comers | Non-experimental - Routine clinical data review | oral PrEP | Operationally |
| Pyra, M^130^ | 2018 | Eastern/Southern Africa | Women (all ages) | Experimental - human subjects | oral PrEP | Operationally |
| Pyra, M^131^ | 2019 | Other: Global | All comers | Review article | not specified | Qualitatively |
| Pyra, M^132^ | 2020 | North America | All comers | Non-experimental - Routine clinical data review | oral PrEP | Operationally |
| Pyra, M^133^ | 2021 | North America | Other: Black ciswomen | Non-experimental - human subjects | oral PrEP | Operationally |
| Pyra, M^134^ | 2022 | North America | Other: Clients who self-identified as Latinx, Asian, or Black | Non-experimental - Routine clinical data review | oral PrEP | Operationally |
| Rao, A^135^ | 2022 | Eastern/Southern Africa | FSW/sex worker | Non-experimental - Routine clinical data review | oral PrEP | Operationally |
| Rao, A^136^ | 2023 | Eastern/Southern Africa | FSW/sex worker; AGYW | Non-experimental - Routine clinical data review | oral PrEP | Operationally |
| Rao, A^137^ | 2023 | Eastern/Southern Africa | FSW/sex worker | Experimental - human subjects | oral PrEP | Operationally |
| Reback, C^26^ | 2024 | North America | MSM; Transgender persons | Other: Protocol paper | oral PrEP; injectable PrEP | Operationally |
| Reisner, S^19^ | 2021 | North America | MSM | Non-experimental - human subjects | oral PrEP; injectable PrEP | Operationally |
| Ridgway, J^138^ | 2023 | North America | Other: Black cisgender women | Other: Protocol paper | oral PrEP | Operationally |
| Riley, T^139^ | 2023 | North America | All comers | Non-experimental - Routine clinical data review | Presumed oral PrEP | Operationally |
| Rolle, C^140^ | 2018 | North America | All comers | Non-experimental - human subjects | oral PrEP | Qualitatively |
| Rousseau, E^37^ | 2021 | Eastern/Southern Africa | AGYW | Non-experimental - human subjects | oral PrEP | Operationally |
| Rousseau, E^141^ | 2023 | Eastern/Southern Africa | AGYW | Experimental - human subjects | oral PrEP | Operationally |
| Rotsaert, A^142^ | 2025 | Eastern/Southern Africa | Pregnant or breastfeeding women | Review article | oral PrEP | Operationally |
| Rugira, E^143^ | 2023 | Eastern/Southern Africa | MSM; FSW/sex worker | Non-experimental - human subjects | oral PrEP | Qualitatively |
| Rutstein, S^144^ | 2022 | Eastern/Southern Africa | All comers | Experimental - human subjects | oral PrEP | Operationally |
| Rutstein, S^22^ | 2025 | Eastern/Southern Africa | Pregnant or breastfeeding women | Experimental - human subjects | oral PrEP; injectable PrEP | Operationally |
| Rutstein, S^145^ | 2025 | Eastern/Southern Africa | All comers | Non-experimental - human subjects | oral PrEP | Operationally |
| Rutstein, S^29^ | 2025 | Eastern/Southern Africa | Heterosexual; Men (all ages) | Other: protocol | oral PrEP; injectable PrEP | Operationally |
| Serota, D^34^ | 2020 | North America | MSM | Non-experimental - human subjects | oral PrEP | Operationally |
| Sevelius, J^146^ | 2024 | Central/South America | Transgender persons | Other: Protocol paper | Presumed oral PrEP | Operationally |
| Shaikh, S^147^ | 2025 | Asia | Transgender persons | Non-experimental - Routine clinical data review | Presumed oral PrEP | Operationally |
| Shangase, N^148^ | 2025 | Eastern/Southern Africa | All comers | Non-experimental - Routine clinical data review | oral PrEP | Operationally |
| Sharpe, J^149^ | 2023 | North America | MSM | Non-experimental - Routine clinical data review | oral PrEP | Operationally |
| Sheth, A^41^ | 2020 | North America | Other: women 13-45 years old | Non-experimental - human subjects | oral PrEP | Operationally |
| Siegler,A^38^ | 2019 | North America | MSM | Experimental - human subjects | Presumed oral PrEP | Qualitatively |
| Spinelli, M^36^ | 2018 | North America | All comers | Non-experimental - Routine clinical data review | Presumed oral PrEP | Operationally |
| Spinelli, M^150^ | 2019 | North America | All comers | Non-experimental - Routine clinical data review | Presumed oral PrEP | Operationally |
| Spinelli, M^151^ | 2020 | North America | MSM | Other: Commentary | not specified | Qualitatively |
| Spinelli, M^152^ | 2020 | North America | All comers | Non-experimental - human subjects | oral PrEP | Qualitatively |
| Spinelli, M^153^ | 2020 | Australia | MSM | Other: Letter to the Editor | Presumed oral PrEP | Qualitatively |
| Stanton, A^31^ | 2022 | Eastern/Southern Africa | Pregnant or breastfeeding women | Review article | oral PrEP; vaginal ring; long-acting PrEP (non-injectable) | Qualitatively |
| Storholm, E^154^ | 2022 | North America | Transgender persons | Experimental - human subjects | oral PrEP | Operationally |
| Storholm, E^155^ | 2022 | North America | MSM | Other: Protocol paper | oral PrEP | Operationally |
| Storholm, E^156^ | 2022 | North America | Transgender persons; Other: Non-binary | Experimental - human subjects | oral PrEP | Operationally |
| Storholm, E^23^ | 2025 | North America | Transgender persons | Experimental - human subjects | oral PrEP; injectable PrEP | Operationally |
| Sullivan, P^157^ | 2025 | North America | All comers | Non-experimental - Routine clinical data review | oral PrEP | Operationally |
| Sutten Coats, C^158^ | 2024 | North America | MSM | Experimental - human subjects | oral PrEP | Qualitatively |
| Tanner, M^159^ | 2020 | North America | Other: Adolescents | Review article | oral PrEP | Qualitatively |
| Tao, J^160^ | 2024 | North America | All comers | Non-experimental - Routine clinical data review | Presumed oral PrEP | Operationally |
| Tapsoba, J^161^ | 2020 | Eastern/Southern Africa | AGYW | Non-experimental - human subjects | oral PrEP | Operationally |
| Tapsoba, J^40^ | 2022 | Eastern/Southern Africa | AGYW | Experimental - human subjects | oral PrEP | Operationally |
| Vanbaelen, T^162^ | 2022 | Europe | All comers | Non-experimental - human subjects | Presumed oral PrEP | Qualitatively |
| Vitruk, O^163^ | 2024 | Europe | All comers | Non-experimental - Routine clinical data review | oral PrEP | Operationally |
| Vu, B^164^ | 2025 | Asia | All comers | Non-experimental - Routine clinical data review | oral PrEP | Operationally |
| Waetjen, M^165^ | 2021 | Europe | Other: "Sexual minority men" | Non-experimental - human subjects | oral PrEP | Operationally |
| Weir, B^166^ | 2023 | Asia | MSM; FSW/sex worker; Transgender persons | Non-experimental - human subjects | oral PrEP | Operationally |
| Wheatley, M^167^ | 2022 | North America | MSM | Other: Network-based HIV transmission model | Presumed oral PrEP | Qualitatively |
| Whelchel, K^168^ | 2023 | North America | All comers | Non-experimental - Routine clinical data review | oral PrEP | Qualitatively |
| Wray, T^169^ | 2023 | North America | Other: "sexual minority men" | Experimental - human subjects | oral PrEP | Qualitatively |
| AGYW: adolescent girls and young women; FSW: female sex worker; MSM: men who have sex with men; PWID: people who inject drugs; STI: sexually transmitted infection | | | | | | |

**Supplemental Table 2.** Studies with operationalizable definitions of PrEP persistence.

| **Author** | **Year** | **Study Population** | **Study Design** | **PrEP Persistence Inputs** | | | | | |
| --- | --- | --- | --- | --- | --- | --- | --- | --- | --- |
|  |  |  |  | **Clinic visit/ refill date** | **Adherence- drug metabolite** | **Adherence - pill count** | **Adherence – patient or participant self-report** | **Self-reported PrEP use**^†^ | **Other** |
| Abuogi, L^50^ | 2025 | Other: Youth 14-24 | Experimental - human subjects | **X** |  |  |  |  |  |
| Argenyi, M^53^ | 2022 | All comers | Non-experimental - Routine clinical data review | **X** |  |  |  |  |  |
| Arnold, T^54^ | 2025 | MSM | Other: Protocol paper | **X** |  |  | **X** |  |  |
| Baeten, J^30^ | 2021 | Women (all ages) | Experimental - human subjects |  | **X** |  |  |  |  |
| Barnabee, G^56^ | 2023 | AGYW | Non-experimental - Routine clinical data review | **X** |  |  |  |  |  |
| Barnabee, G^55^ | 2022 | AGYW | Experimental - human subjects | **X** |  |  |  |  |  |
| Bassett, I^57^ | 2026 | AGYW | Experimental - human subjects | **X** |  |  |  |  |  |
| Bazzi, A^18^ | 2023 | PWID | Experimental - human subjects | **X** |  |  |  |  |  |
| Bien-Gund, C^58^ | 2022 | Women (all ages) | Experimental - human subjects |  |  |  |  | **X** |  |
| Blumenthal, J^59^ | 2021 | Women (all ages) | Experimental - human subjects |  |  |  |  | **X** |  |
| Bogart, L^60^ | 2024 | All comers | Experimental - human subjects | **X** |  | **X** |  |  |  |
| Braun, R^61^ | 2023 | MSM; Transgender persons | Experimental - human subjects |  |  |  | **X** |  |  |
| Bruxvoort, K^62^ | 2021 | All comers | Non-experimental - Routine clinical data review | **X** |  |  |  |  |  |
| Bui, H^27^ | 2024 | MSM | Other: protocol paper | X |  |  |  |  |  |
| Celum, C^68^ | 2023 | AGYW | Experimental - human subjects | **X** |  |  |  |  |  |
| Celum, C^67^ | 2022 | AGYW | Non-experimental - human subjects | **X** |  |  |  |  |  |
| Celum, C^66^ | 2021 | AGYW | Experimental - human subjects | **X** | **X** |  |  |  |  |
| Celum, C^65^ | 2020 | AGYW | Experimental - human subjects | **X** |  |  |  |  |  |
| Chan, P^69^ | 2025 | MSM | Non-experimental - human subjects | **X** |  |  |  |  |  |
| Chapin-Bardales, J^70^ | 2023 | MSM | Non-experimental - human subjects |  | **X** |  | **X** |  |  |
| Chase, E^71^ | 2022 | All comers | Non-experimental - Routine clinical data review | **X** |  |  |  |  |  |
| Chen, Y^72^ | 2024 | MSM | Non-experimental - Routine clinical data review | **X** |  |  |  |  |  |
| Chipukuma, J^74^ | 2023 | AGYW | Non-experimental - Routine clinical data review | **X** |  |  |  |  |  |
| Clement, M^76^ | 2021 | All comers | Non-experimental - Routine clinical data review | **X** |  |  |  |  |  |
| Clement, M^75^ | 2019 | All comers | Non-experimental - Routine clinical data review | **X** |  |  |  |  |  |
| Colver, J^77^ | 2025 | Other: active-duty military | Non-experimental - Routine clinical data review | **X** |  |  |  |  |  |
| Coy, K^39^ | 2019 | All comers | Non-experimental - Routine clinical data review | **X** |  |  |  |  |  |
| Davey, J^45^I | 2022 | Pregnant or breastfeeding women | Other: protocol paper |  | **X** | **X** | **X** |  |  |
| Davey, D^78^ | 2025 | AGYW; Men (all ages); Other: young people | Experimental - human subjects | **X** |  |  |  |  |  |
| de Voux, A^79^ | 2023 | Pregnant or breastfeeding women | Experimental - human subjects | **X** |  |  |  |  |  |
| Dean, L^80^ | 2021 | All comers | Non-experimental - Routine clinical data review | **X** |  |  |  |  |  |
| Doblecki-Lewis, S^81^ | 2024 | All comers | Non-experimental - Routine clinical data review | **X** |  |  |  |  |  |
| Fan, C^83^ | 2025 | MSM | Experimental - human subjects | **X** |  |  |  |  |  |
| Felker-Kantor, E^84^ | 2023 | Men (all ages) | Non-experimental - Routine clinical data review | **X** |  |  |  |  |  |
| Fischer, A^85^ | 2025 | AGYW | Review article | **X** |  |  |  |  |  |
| Gichane, M^87^ | 2024 | AGYW | Experimental - human subjects |  | **X** |  |  |  |  |
| Goedel, W^90^ | 2022 | MSM | Experimental - human subjects | **X** |  |  |  | **X** |  |
| Haribhai, S^93^ | 2023 | Other: pregnant AGYW | Non-experimental - human subjects |  |  |  | **X** |  |  |
| Heffron, R^94^ | 2025 | Women (all ages) | Experimental - human subjects | **X** |  |  |  |  |  |
| Hendrickson, C^44^ | 2020 | Not specified | Review article | **X** |  |  |  |  |  |
| Hendrickson, C | 2025 | MSM; FSW/sex worker; AGYW | Other: cost-effectiveness | **X** |  |  |  |  |  |
| Hill, N | 2025 | Other: Youth aged 18-25 | Non-experimental - Routine clinical data review | **X** |  |  |  |  |  |
| Holloway, I^98^ | 2020 | MSM | Non-experimental - human subjects |  |  |  |  | **X** |  |
| Huang, YL^35^ | 2021 | All comers | Non-experimental - Routine clinical data review | **X** |  |  |  |  |  |
| Isehunwa, O^99^ | 2024 | Pregnant or breastfeeding women; Women (all ages) | Non-experimental - human subjects |  |  |  |  | **X** |  |
| Jin, E^24^ | 2025 | AGYW | Other: Modeling study | **X** |  |  |  |  |  |
| John, S^104^ | 2019 | MSM | Non-experimental - human subjects |  |  |  | **X** |  | **X**^‡^ |
| Jones, J^105^ | 2022 | MSM | Non-experimental - human subjects |  |  |  |  | **X** |  |
| Keyes, J^107^ | 2020 | All comers | Non-experimental - Routine clinical data review | **X** |  |  |  |  |  |
| Khadka, N^108^ | 2023 | Other: Pregnant and postpartum AGYW | Non-experimental - human subjects |  | **X** |  |  |  |  |
| Khati, A^109^ | 2025 | PWID | Experimental - human subjects | **X** |  |  |  |  |  |
| Kumar, R^28^ | 2024 | FSW/sex worker | Other: Protocol paper | **X** |  | **X** |  |  |  |
| Kusemererwa, S^20^ | 2025 | Men (all ages) | Experimental - human subjects | **X** | **X** |  |  |  |  |
| Lagat, H^32^ | 2024 | AGYW | Other: Protocol paper |  |  |  |  | **X** |  |
| Le Roux, C^112^ | 2024 | All comers | Non-experimental - Routine clinical data review | **X** |  |  |  | **X** |  |
| Matsuno, A^114^ | 2025 | AGYW | Non-experimental - human subjects | **X** |  |  |  | **X** |  |
| Matthews, L^115^ | 2024 | Women (all ages) | Non-experimental - human subjects |  | **X** |  |  |  |  |
| McCormick, C^116^ | 2023 | All comers | Non-experimental - Routine clinical data review | **X** |  |  |  |  |  |
| Mirembe, B^118^ | 2024 | AGYW | Experimental - human subjects | **X** |  |  |  |  |  |
| Moon, E^119^ | 2025 | All comers | Non-experimental - Routine clinical data review | **X** |  |  |  |  |  |
| Mulholland, G^120^ | 2025 | All comers | Non-experimental - Routine clinical data review | **X** |  |  |  |  |  |
| Oglesby, A^123^ | 2024 | All comers | Non-experimental - Routine clinical data review | **X** |  |  |  |  |  |
| Onwubiko, U^125^ | 2024 | MSM | Non-experimental - human subjects |  |  |  |  | **X** |  |
| O'Rourke, S^126^ | 2021 | AGYW | Experimental - human subjects |  | **X** |  |  |  |  |
| Pike, C^21^ | 2025 | Other: young people 15-29 | Other: Protocol paper | **X** |  |  |  |  |  |
| Pintye, J | 2023 | Pregnant or breastfeeding women | Experimental - human subjects |  |  |  | **X** |  |  |
| Platt, L^129^ | 2023 | All comers | Non-experimental - Routine clinical data review | **X** |  |  |  |  |  |
| Pyra, M^134^ | 2022 | Other: Clients who self-identified as Latinx, Asian, or Black | Non-experimental - Routine clinical data review | **X** |  | **X** |  |  |  |
| Pyra, M^133^ | 2021 | Other: Black ciswomen | Non-experimental - human subjects | **X** |  |  |  |  |  |
| Pyra, M^132^ | 2020 | All comers | Non-experimental - Routine clinical data review | **X** | **X** |  |  |  |  |
| Pyra, M^130^ | 2018 | Women (all ages) | Experimental - human subjects |  |  | **X** |  |  |  |
| Rao, A^137^ | 2023 | FSW/sex worker | Experimental - human subjects | **X** |  |  |  |  |  |
| Rao, A^136^ | 2023 | FSW/sex worker; AGYW | Non-experimental - Routine clinical data review | **X** |  | **X** |  |  |  |
| Rao, A^135^ | 2022 | FSW/sex worker | Non-experimental - Routine clinical data review | **X** |  |  |  |  |  |
| Reback, C^26^ | 2024 | MSM; Transgender persons | Other: Protocol paper | **X** | **X** |  |  |  |  |
| Reisner, S^19^ | 2021 | MSM | Non-experimental - human subjects |  |  |  |  | **X** |  |
| Ridgway, J^138^ | 2023 | Other: Black cisgender women | Other: Protocol paper | **X** |  |  |  |  |  |
| Riley, T^139^ | 2024 | All comers | Non-experimental - Routine clinical data review | **X** |  |  |  |  |  |
| Rolle, C^140^ | 2018 | All comers | Non-experimental - human subjects | **X** |  |  |  |  |  |
| Rotsaert, A^142^ | 2025 | Pregnant or breastfeeding women | Review article | **X** |  |  |  | **X** |  |
| Rousseau, E^141^ | 2023 | AGYW | Experimental - human subjects | **X** |  |  |  |  |  |
| Rousseau, E^37^ | 2021 | AGYW | Non-experimental - human subjects | **X** |  |  |  |  |  |
| Rutstein, S^144^ | 2022 | All comers | Experimental - human subjects | **X** |  | **X** |  |  |  |
| Rutstein, S^22^ | 2025 | Pregnant or breastfeeding women | Experimental - human subjects | **X** |  |  |  |  |  |
| Rutstein, S^145^ | 2025 | All comers | Non-experimental - human subjects | **X** |  | **X** |  |  |  |
| Rutstein, S^29^ | 2025 | Heterosexual; Men (all ages) | Other: protocol | **X** | **X** |  | **X** |  |  |
| Serota, D^34^ | 2020 | MSM | Non-experimental - human subjects | **X** |  |  | **X** |  |  |
| Sevelius, J^146^ | 2024 | Transgender persons | Other: Protocol paper | **X** |  |  |  |  |  |
| Shaikh, S^147^ | 2025 | Transgender persons | Non-experimental - Routine clinical data review | **X** |  |  |  |  |  |
| Shangase, N^148^ | 2025 | All comers | Non-experimental - Routine clinical data review | **X** |  |  |  |  |  |
| Sharpe, J^149^ | 2023 | MSM | Non-experimental - Routine clinical data review |  |  |  |  | **X** |  |
| Sheth, A^41^ | 2020 | Other: women 13-45 years old | Non-experimental - human subjects | **X** |  |  |  |  |  |
| Spinelli, M^150^ | 2019 | All comers | Non-experimental - Routine clinical data review | **X** |  |  |  |  |  |
| Spinelli, M^36^ | 2018 | All comers | Non-experimental - Routine clinical data review | **X** |  |  |  |  |  |
| Storholm, E^155^ | 2022 | MSM | Other: Protocol paper |  | **X** |  |  | **X** |  |
| Storholm, E^156^ | 2022 | Transgender persons; Other: Non-binary | Experimental - human subjects |  | **X** |  |  |  |  |
| Storholm, E^154^ | 2022 | Transgender persons | Experimental - human subjects |  | **X** |  |  |  |  |
| Storholm, E^23^ | 2025 | Transgender persons | Experimental - human subjects | **X** | **X** |  |  |  |  |
| Sullivan, P^157^ | 2025 | All comers | Non-experimental - Routine clinical data review | **X** |  |  |  |  |  |
| Tao, J^160^ | 2024 | All comers | Non-experimental - Routine clinical data review | **X** |  |  |  |  |  |
| Tapsoba, J^40^ | 2022 | AGYW | Experimental - human subjects | **X** |  |  |  |  | **X**^§^ |
| Tapsoba, J^161^ | 2020 | AGYW | Non-experimental - human subjects | **X** |  |  |  |  |  |
| Vitruk, O^163^ | 2024 | All comers | Non-experimental - Routine clinical data review | **X** |  |  |  |  |  |
| Vu, B^164^ | 2025 | All comers | Non-experimental - Routine clinical data review | **X** |  |  |  |  |  |
| Waetjen, M^165^ | 2021 | Other: "Sexual minority men" | Non-experimental - human subjects |  |  |  |  |  | **X**^¶^ |
| Weir, B^166^ | 2023 | MSM; FSW/sex worker; Transgender persons | Non-experimental - human subjects |  |  |  |  | **X** |  |
| ^†^ Patient or participant self-report of any PrEP use at time of assessment or within specific recall window  ^‡^ Quarterly HIV or STI testing ^§^ Intent to use PrEP  ^¶^ Supply of PrEP during 12-month period (PrEP availability)  AGYW: adolescent girls and young women; FSW: female sex worker; MSM: men who have sex with men; PWID: people who inject drugs; STI: sexually transmitted infection | | | | | | | | | |

**References**

50. Abuogi LL, Imran R, Limas A, et al. Pilot outcomes of a telehealth model for youth PrEP (TelePrEP) among youth at risk for HIV in Colorado. *HIV Res Clin Pract*. 2025;26(1):2588009. doi:10.1080/25787489.2025.2588009

51. Amico KR, Wallace M, Bekker LG, et al. Experiences with HPTN 067/ADAPT Study-Provided Open-Label PrEP Among Women in Cape Town: Facilitators and Barriers Within a Mutuality Framework. *AIDS Behav*. 2017;21(5):1361-1375. doi:10.1007/s10461-016-1458-y

52. Amico KR, Stirratt MJ. Adherence to preexposure prophylaxis: Current, emerging, and anticipated bases of evidence. *Clin Infect Dis*. 2014;59 Suppl 1(SUPPL.1):S55-S60. doi:10.1093/cid/ciu266

53. Argenyi MS, Molotnikov LE, Leach DH, Roosevelt KA, Klevens RM, Hsu KK. Gaps in HIV preexposure prophylaxis continuum of care following state partner services for Massachusetts primary and secondary syphilis cases, 2017 to 2018. *Sex Transm Dis*. 2022;49(9):657-661. doi:10.1097/OLQ.0000000000001669

54. Arnold T, Giorlando KK, Barnett AP, et al. Acceptance-based pre-exposure prophylaxis intervention (ACTPrEP) to engage young Black men who have sex with men in the southern United States: Protocol for a pilot randomized controlled trial. *JMIR Res Protoc*. 2025;14:e65921. doi:10.2196/65921

55. Barnabee G, O’Bryan G, Ndeikemona L, et al. Improving HIV pre-exposure prophylaxis persistence among adolescent girls and young women: Insights from a mixed-methods evaluation of community, hybrid, and facility service delivery models in Namibia. *Front Reprod Health*. 2022;4:1048702. doi:10.3389/frph.2022.1048702

56. Barnabee G, Billah I, Ndeikemona L, et al. Prep uptake and early persistence among adolescent girls and young women receiving services via community and hybrid community-clinic models in Namibia. *PLoS One*. 2023;18(8 August):e0289353. doi:10.1371/journal.pone.0289353

57. Bassett IV, Yan J, Govere S, et al. PrEP and contraceptive persistence among young women receiving sexual and reproductive health services through hair salons in South Africa. *J Acquir Immune Defic Syndr*. 2026;101(3):288-295. doi:10.1097/QAI.0000000000003800

58. Bien-Gund CH, Ochwal P, Marcus N, et al. Adoption of HIV pre-exposure prophylaxis among women at high risk of HIV infection in Kenya. *PLoS One*. 2022;17(9 9):e0273409. doi:10.1371/journal.pone.0273409

59. Blumenthal J, Jain S, He F, et al. Results from a pre-exposure prophylaxis demonstration project for at-risk cisgender women in the United States. *Clin Infect Dis*. 2021;73(7):1149-1156. doi:10.1093/cid/ciab328

60. Bogart LM, Musoke W, Mukama CS, et al. Enhanced oral pre-exposure prophylaxis (PrEP) implementation for Ugandan fisherfolk: Pilot intervention outcomes. *AIDS Behav*. 2024;28(10):3512-3524. doi:10.1007/s10461-024-04432-w

61. Braun RA, Erenrich RK, Coyle KK, Doan THP, Klausner JD. Effectiveness, Acceptability, and Feasibility of a Telehealth HIV Pre-Exposure Prophylaxis Care Intervention Among Young Cisgender Men and Transgender Women Who Have Sex With Men: Protocol for a Randomized Controlled Trial. *JMIR Res Protoc*. 2023;12:e47932. doi:10.2196/47932

62. Bruxvoort KJ, Schumacher CM, Towner W, et al. Referral Linkage to Preexposure Prophylaxis Care and Persistence on Preexposure Prophylaxis in an Integrated Health Care System. *J Acquir Immune Defic Syndr*. 2021;87(3):918-927. doi:10.1097/QAI.0000000000002668

63. Butts SA, Johnson AL, Doblecki-Lewis S. PrEP beyond the clinic: Evaluation of a home-based PrEP follow-up system among a cohort of predominantly black and Latino men who have sex with men in South Florida. *J Acquir Immune Defic Syndr*. Published online March 28, 2025. doi:10.1097/QAI.0000000000003664

64. Celum CL, Delany-Moretlwe S, Baeten JM, et al. HIV pre-exposure prophylaxis for adolescent girls and young women in Africa: from efficacy trials to delivery. *J Int AIDS Soc*. 2019;22 Suppl 4(Suppl Suppl 4):e25298. doi:10.1002/jia2.25298

65. Celum CL, Gill K, Morton JF, et al. Incentives conditioned on tenofovir levels to support PrEP adherence among young South African women: a randomized trial. *J Int AIDS Soc*. 2020;23(11):e25636. doi:10.1002/jia2.25636

66. Celum C, Hosek S, Tsholwana M, et al. PrEP uptake, persistence, adherence, and effect of retrospective drug level feedback on PrEP adherence among young women in southern Africa: Results from HPTN 082, a randomized controlled trial. *PLoS Med*. 2021;18(6):e1003670. doi:10.1371/journal.pmed.1003670

67. Celum CL, Bukusi EA, Bekker LG, et al. PrEP use and HIV seroconversion rates in adolescent girls and young women from Kenya and South Africa: the POWER demonstration project. *J Int AIDS Soc*. 2022;25(7):e25962. doi:10.1002/jia2.25962

68. Celum C, Seidman D, Travill D, et al. A decision support tool has similar high PrEP uptake and increases early prep persistence in adolescent girls and young women in South Africa: results from a randomized controlled trial. *J Int AIDS Soc*. 2023;26(8):e26154. doi:10.1002/jia2.26154

69. Chan PA, Goedel WC, Li Y, et al. Impact of social determinants of health on pre-exposure prophylaxis care for HIV prevention. *J Acquir Immune Defic Syndr*. 2025;98(5):465-472. doi:10.1097/QAI.0000000000003601

70. Chapin-Bardales J, Haaland R, Martin A, et al. HIV pre-exposure prophylaxis persistence and adherence among men Who have sex with men in four US cities. *J Acquir Immune Defic Syndr Hum Retrovirol*. 2023;93(1):34-41. doi:10.1097/QAI.0000000000003160

71. Chase E, Mena L, Johnson KL, Prather M, Khosropour CM. Patterns of Pre-exposure Prophylaxis (PrEP) Use in a Population Accessing PrEP in Jackson, Mississippi. *AIDS Behav*. 2022;27(4):1082-1090. doi:10.1007/s10461-022-03845-9

72. Chen YN, Zhou J, Kirkham HS, et al. Understanding typology of preexposure prophylaxis (PrEP) persistence trajectories among male PrEP users in the United States. *Open Forum Infect Dis*. 2024;11(11):ofae584. doi:10.1093/ofid/ofae584

73. Chen-Charles J, Joseph Davey D, Toska E, Seeley J, Bekker LG. PrEP Uptake and Utilisation Among Adolescent Girls and Young Women in Sub-Saharan Africa: A Scoping Review. AIDS Behav. 2025 Jun;29(6):1876-1896. doi: 10.1007/s10461-025-04656-4.

74. Chipukuma J, Lindsay B, Mwango LK, et al. FOSTERING ACCESS TO PREP AMONG ADOLESCENT GIRLS AND YOUNG WOMEN AGED 16 TO 24 YEARS AT HIGH RISK OF HIV THROUGH THE DREAMS INITIATIVE IN FOUR DISTRICTS IN ZAMBIA. *AIDS Educ Prev*. 2023;35:52-66. doi:10.1521/aeap.2023.35.suppA.52

75. Clement ME, Johnston BE, Eagle C, et al. Advancing the HIV pre-exposure prophylaxis continuum: a collaboration between a public health department and a federally qualified health center in the southern United States. *AIDS Patient Care STDS*. 2019;33(8):366-371. doi:10.1089/apc.2019.0054

76. Clement ME, Nicchitta M, Sun Y, et al. Preexposure Prophylaxis Outcomes in an Urban Community in North Carolina: Discontinuation of Care and Sexually Transmitted Infections. *Sex Transm Dis*. 2021;48(3):183-188. doi:10.1097/OLQ.0000000000001288

77. Colver JT, Yabes JM Jr, Marcus JE. Persistence with Human Immunodeficiency Virus Pre-exposure Prophylaxis in an active-duty military population. *AIDS Behav*. 2025;29(2):607-612. doi:10.1007/s10461-024-04543-4

78. Joseph Davey D, Fynn L, Rousseau E, et al. Evaluation of point-of-care diagnostics for sexually transmitted infection on oral PrEP initiation and persistence among young people in South Africa: a randomized controlled study. *J Int AIDS Soc*. 2025;28(5):e26488. doi:10.1002/jia2.26488

79. De Voux A, Silliman M, Mvududu R, Mashele N, Myer L, Davey DJ. RANDOMIZED EVALUATION OF THE IMPACT OF STI POINT-OF-CARE TESTING ON PrEP INITIATION. *Top Antivir Med*. 2023;31(2):388. https://www.embase.com/search/results?subaction=viewrecord&id=L641190513&from=export

80. Dean LT, Chang HY, Goedel WC, Chan PA, Doshi JA, Nunn AS. Novel population-level proxy measures for suboptimal HIV preexposure prophylaxis initiation and persistence in the USA. *AIDS*. 2021;35(14):2375-2381. doi:10.1097/QAD.0000000000003030

81. Doblecki-Lewis S, Johnson A, Klose K, et al. An observational cohort study evaluating PrEP reach, engagement and persistence through a community-based mobile clinic in Miami-Dade County, Florida. *J Int AIDS Soc*. 2024;27(10):e26362. doi:10.1002/jia2.26362

82. Evans KN, Hassan R, Townes A, Buchacz K, Smith DK. The Potential of Telecommunication Technology to Address Racial/Ethnic Disparities in HIV PrEP Awareness, Uptake, Adherence, and Persistence in Care: A Review. *AIDS Behav*. 2022;26(12):3878-3888. doi:10.1007/s10461-022-03715-4

83. Fan C, Yin Z, Li C, et al. Impact of peer referral on pre-exposure prophylaxis adherence and persistence among men who have sex with men: A cohort study in China. *AIDS Behav*. 2025;29(6):1831-1840. doi:10.1007/s10461-025-04651-9

84. Felker-Kantor E, Greener LR, Mabaso S, et al. Understanding Oral PrEP Interest, Uptake, Persistence, and Experience of Use Among Heterosexual Men in Johannesburg, South Africa: An Exploratory Pilot Study. *AIDS Behav*. 2023;28(2):564-573. doi:10.1007/s10461-023-04246-2

85. Fischer AE, Hanif H, Stocks JB, et al. Mobile health intervention tools promoting HIV pre-exposure prophylaxis among adolescent girls and young women in sub-Saharan Africa: Scoping review. *JMIR MHealth UHealth*. 2025;13:e60819. doi:10.2196/60819

86. Furukawa NW, Schneider JA, Coleman ME, Wiener JB, Shrestha RK, Smith DK. Out-of-pocket costs and HIV pre-exposure prophylaxis persistence in a US multicity demonstration project. *Health Serv Res*. 2020;55(4):524-530. doi:10.1111/1475-6773.13285

87. Gichane MW, Velloza J, Hosek S, et al. Hoping to adhere? Examining the relationship between hope and pre-exposure prophylaxis willingness, adherence, and persistence among young women in South Africa and Zimbabwe (HPTN 082). *AIDS Behav*. 2025;29(2):527-534. doi:10.1007/s10461-024-04536-3

88. Gillespie D, Wood F, Williams A, et al. Experiences of men who have sex with men when initiating, implementing and persisting with HIV pre-exposure prophylaxis. *Health Expect*. 2022;25(4):1332-1341. doi:10.1111/hex.13446

89. Goedel WC, Rogers BG, Li Y, et al. Pre-exposure Prophylaxis Discontinuation During the COVID-19 Pandemic Among Men Who Have Sex With Men in a Multisite Clinical Cohort in the United States. *J Acquir Immune Defic Syndr*. 2022;91(2):151-156. doi:10.1097/QAI.0000000000003042

90. Goedel WC, Coats CS, Chan PA, et al. A pilot study of a patient navigation intervention to improve HIV pre-exposure prophylaxis persistence among Black/African American men who have sex with men. *J Acquir Immune Defic Syndr Hum Retrovirol*. 2022;90(3):276-282. doi:10.1097/QAI.0000000000002954

91. Haberer JE, Mujugira A, Mayer KH. The future of HIV pre-exposure prophylaxis adherence: reducing barriers and increasing opportunities. *Lancet HIV*. 2023;10(6):e404-e411. doi:10.1016/S2352-3018(23)00079-6

92. Xavier Hall CD, Bundy C, Foran JE, et al. Identifying Strategies for Improving Pre-exposure Prophylaxis Adherence: Perspectives from a Sample of Highly Adherent Young Men Who have Sex with Men. *AIDS Behav*. 2022;27(2):506-517. doi:10.1007/s10461-022-03785-4

93. Haribhai S, Khadka N, Mvududu R, et al. Psychosocial determinants of pre-exposure prophylaxis use among pregnant adolescent girls and young women in Cape Town, South Africa: a qualitative study. *Int J STD AIDS*. 2023;34(8):548-556. doi:10.1177/09564624231152776

94. Heffron R, Etyang L, Nyerere B, et al. Phone calls to enhance PrEP persistence among Kenyan women accessing postabortal care: a cluster randomized trial. *Front Reprod Health*. 2025;7(1709721):1709721. doi:10.3389/frph.2025.1709721

95. Hendrickson C, Hirasen K, Mongwenyana C, et al. Costs and outcomes of routine HIV oral pre-exposure prophylaxis implementation across different service delivery models and key populations in South Africa: a retrospective cohort study. *Lancet HIV*. 2025;12(2):e130-e142. doi:10.1016/S2352-3018(24)00295-9

96. Hill NE, He S, Chua KP. National trends in HIV pre-exposure prophylaxis dispensing to young adults, 2016-2023. *J Gen Intern Med*. 2026;41(1):53-63. doi:10.1007/s11606-025-09574-8

97. Hodges-Mameletzis I, Fonner VA, Dalal S, Mugo N, Msimanga-Radebe B, Baggaley R. Pre-exposure prophylaxis for HIV prevention in women: current status and future directions. *Drugs*. 2019;79(12):1263-1276. doi:10.1007/s40265-019-01143-8

98. Holloway IW, Krueger EA, Meyer IH, Lightfoot M, Frost DM, Hammack PL. Longitudinal trends in PrEP familiarity, attitudes, use and discontinuation among a national probability sample of gay and bisexual men, 2016-2018. *PLoS One*. 2020;15(12 December 2020):e0244448. doi:10.1371/journal.pone.0244448

99. Isehunwa OO, Jaggernath M, Kriel Y, et al. Uptake and persistence of safer conception strategies among South African women planning for pregnancy. *AIDS Behav*. 2024;28(12):4029-4039. doi:10.1007/s10461-024-04475-z

100. Jackson-Gibson M, Ezema AU, Orero W, et al. Facilitators and barriers to HIV pre-exposure prophylaxis (PrEP) uptake through a community-based intervention strategy among adolescent girls and young women in Seme Sub-County, Kisumu, Kenya. *BMC Public Health*. 2021;21(1284):1284. doi:10.1186/s12889-021-11335-1

101. Jenness SM, Knowlton G, Smith DK, et al. A decision analytics model to optimize investment in interventions targeting the HIV preexposure prophylaxis cascade of care. *AIDS*. 2021;35(9):1479-1489. doi:10.1097/QAD.0000000000002909

102. Jiang H, Zou H. PrEParing for HIV prevention among men who have sex with men in China: challenges and solutions. *Lancet Glob Health*. 2025;13(9):e1636-e1641. doi:10.1016/S2214-109X(25)00234-7

103. John SA, Rendina HJ, Grov C, Parsons JT. Home-based pre-exposure prophylaxis (PrEP) services for gay and bisexual men: an opportunity to address barriers to PrEP uptake and persistence. *PLoS One*. 2017;12(12):e0189794. doi:10.1371/journal.pone.0189794

104. John SA, Rendina HJ, Starks TJ, Grov C, Parsons JT. Decisional balance and contemplation ladder to support interventions for HIV pre-exposure prophylaxis uptake and persistence. *AIDS Patient Care STDS*. 2019;33(2):67-78. doi:10.1089/apc.2018.0136

105. Jones J, Pampati S, Siegler AJ. Alignment of PrEP use and sexual behavior over four months among men who have sex with men in the southern United States. *AIDS Behav*. 2022;26(10):3378-3385. doi:10.1007/s10461-022-03685-7

106. Jones J, Pampati S, Emrick K, Siegler AJ. Demographic and behavioral characteristics of urban and non-urban PrEP-using MSM in the South. *AIDS Care*. 2022;34(11):1461-1464. doi:10.1080/09540121.2022.2085864

107. Keyes J, Crouse EC, DeJesus E, Rolle CP. Determinants of pre-exposure prophylaxis (PrEP) persistence in a high-risk population in Central Florida. *J Investig Med*. 2020;69(2):397-401. doi:10.1136/jim-2020-001352

108. Khadka N, Gorbach PM, Nyemba DC, et al. Evaluating the use of oral pre-exposure prophylaxis among pregnant and postpartum adolescent girls and young women in Cape Town, South Africa. *Front Reprod Health*. 2023;5:1224474. doi:10.3389/frph.2023.1224474

109. Khati A, Copenhaver M, Xu R, et al. Oral pre-exposure prophylaxis (PrEP) receipt and persistence among opioid-dependent people who inject drugs initiating PrEP for HIV prevention. *J Subst Use Addict Treat*. 2025;173(209693):209693. doi:10.1016/j.josat.2025.209693

110. Khosropour CM, Riley T, Healy E, et al. Persistence in a pharmacist-led, same-day PrEP program in Mississippi: a mixed-methods study. *BMC Public Health*. 2023;23(1130):1130. doi:10.1186/s12889-023-16072-1

111. Laborde ND, Kinley PM, Spinelli M, et al. Understanding PrEP Persistence: Provider and Patient Perspectives. *AIDS Behav*. 2020;24(9):2509-2519. doi:10.1007/s10461-020-02807-3

112. Le Roux C, Tassi MF, Faussat C, et al. Factors associated with PrEP persistence and loss of follow-up: A 5-year historic cohort. *Arch Sex Behav*. 2024;53(7):2445-2452. doi:10.1007/s10508-024-02862-0

113. Lelutiu-Weinberger C, Filimon ML, Zavodszky AM, et al. Prepare Romania: study protocol for a randomized controlled trial of an intervention to promote pre-exposure prophylaxis adherence and persistence among gay, bisexual, and other men who have sex with men. *Trials*. 2024;25(1):470. doi:10.1186/s13063-024-08313-4

114. Matsuno A, Tollefson D, Cover J, et al. What drives adolescent girls and young women’s decisions to persist on PrEP? Results of a comparative qualitative study from a DREAMS program in western Kenya. *AIDS Care*. Published online October 13, 2025:1-11. doi:10.1080/09540121.2025.2570098

115. Matthews LT, Jaggernath M, Kriel Y, et al. Oral preexposure prophylaxis uptake, adherence, and persistence during periconception periods among women in South Africa. *AIDS*. 2024;38(9):1342-1354. doi:10.1097/QAD.0000000000003925

116. McCormick CD, Sullivan PS, Qato DM, Crawford SY, Schumock GT, Lee TA. Adherence and persistence of HIV pre-exposure prophylaxis use in the United States. *Pharmacoepidemiol Drug Saf*. 2023;33(1):e5729. doi:10.1002/pds.5729

117. McNulty MC, Kerman J, Devlin SA, et al. Prep persistence support and monitoring in areas of high HIV burden in the midwestern United States. *AIDS Educ Prev*. 2023;35(3):235-246. doi:10.1521/aeap.2023.35.3.235

118. Gati Mirembe B, Donnell D, Krows M, et al. High recent PrEP adherence with point-of-care urine tenofovir testing and adherence counselling among young African women: results from the INSIGHT cohort. *J Int AIDS Soc*. 2024;27(12):e26389. doi:10.1002/jia2.26389

119. Moon EG, Ihnatiuk AP, Kazanzhy AP, et al. HIV pre-exposure prophylaxis (PrEP) uptake and persistence in wartime Ukraine: Analysis of data from a scaled PrEP program. *AIDS Behav*. 2025;29(10):3253-3263. doi:10.1007/s10461-025-04773-0

120. Mulholland GE, Matoga M, Chen JS, et al. Poor oral HIV pre-exposure prophylaxis (PrEP) persistence in an integrated PrEP/STI program in Malawi. *AIDS Behav*. Published online November 29, 2025. doi:10.1007/s10461-025-04937-y

121. Naz-McLean S, Clark J, Huerta L, et al. Social, economic, and physical side effects impact PrEP uptake and persistence among transgender women in Peru. *BMC Public Health*. 2024;24(1):1985. doi:10.1186/s12889-024-19474-x

122. Ndimande-Khoza MN, Katz AWK, Moretlwe-Delany S, et al. Family influences on oral prep use among adolescent girls and young women in Kenya and South Africa. *PLoS One*. 2023;18(11vember):e0292529. doi:10.1371/journal.pone.0292529

123. Oglesby A, Germain G, Metzner AA, et al. Pre-exposure prophylaxis for the prevention of HIV-1: An assessment of oral pre-exposure prophylaxis usage patterns, first evidence of HIV-1, and HIV-1 risk factors in the United States. *AIDS Patient Care STDS*. 2024;38(11):495-506. doi:10.1089/apc.2024.0158

124. Ohiomoba RO, Owuor PM, Orero W, et al. Pre-Exposure Prophylaxis (PrEP) Initiation and Retention Among Young Kenyan Women. *AIDS Behav*. 2022;26(7):2376-2386. doi:10.1007/s10461-022-03576-x

125. Onwubiko UN, Murray SM, Rao A, et al. Individual & joint associations of sexual stigma and mental distress with PrEP uptake, adherence and persistence among US gay and bisexual men. *Soc Sci Med*. 2024;363(117493):117493. doi:10.1016/j.socscimed.2024.117493

126. O’Rourke S, Hartmann M, Myers L, et al. The PrEP Journey: Understanding How Internal Drivers and External Circumstances Impact The PrEP Trajectory of Adolescent Girls and Young Women in Cape Town, South Africa. *AIDS Behav*. 2021;25(7):2154-2165. doi:10.1007/s10461-020-03145-0

127. Pintye J, O’Malley G, Kinuthia J, et al. Influences on early discontinuation and persistence of daily oral PrEP use among Kenyan adolescent girls and young women: a qualitative evaluation from a PrEP implementation program. *J Acquir Immune Defic Syndr Hum Retrovirol*. 2021;86(4):e83-e89. doi:10.1097/QAI.0000000000002587

128. Pintye J, Kinuthia J, Abuna F, et al. HIV pre-exposure prophylaxis initiation, persistence, and adherence during pregnancy through the postpartum period. *AIDS*. 2023;37(11):1725-1737. doi:10.1097/QAD.0000000000003617

129. Platt L, Shebl FM, Qian Y, et al. Pre-exposure Prophylaxis Persistence at a Diverse Sexual Health Clinic: Comparison of the pre-COVID-19 era to the COVID-19 era. *AIDS Behav*. 2023;27(8):2731-2740. doi:10.1007/s10461-023-03996-3

130. Pyra M, Haberer JE, Heffron R, et al. PrEP use during periods of HIV risk among East African women in serodiscordant relationships. *J Acquir Immune Defic Syndr Hum Retrovirol*. 2018;77(1):41-45. https://search.ebscohost.com/login.aspx?direct=true&db=lhh&AN=20183222343&site=ehost-live&scope=site

131. Pyra MN, Haberer JE, Hasen N, Reed J, Mugo NR, Baeten JM. Global implementation of PrEP for HIV prevention: setting expectations for impact. *J Int AIDS Soc*. 2019;22(8):e25370. doi:10.1002/jia2.25370

132. Pyra M, Rusie L, Castro M, et al. A taxonomy of pragmatic measures of HIV preexposure prophylaxis use. *AIDS*. 2020;34(13):1951-1957. doi:10.1097/QAD.0000000000002618

133. Pyra M, Johnson AK, Devlin S, et al. HIV Pre-exposure Prophylaxis Use and Persistence among Black Ciswomen: “Women Need to Protect Themselves, Period.” *J Racial Ethn Health Disparities*. 2021;9(3):820-829. doi:10.1007/s40615-021-01020-9

134. Pyra M, Brewer R, Rusie L, Kline J, Schneider J, Willis I. Long-term HIV pre-exposure prophylaxis trajectories among racial & ethnic minority patients: short, declining, & sustained adherence. *J Acquir Immune Defic Syndr Hum Retrovirol*. 2022;89(2):166-171. doi:10.1097/QAI.0000000000002833

135. Rao A, Mhlophe H, Comins C, et al. Persistence on oral pre-exposure prophylaxis (PrEP) among female sex workers in eThekwini, South Africa, 2016-2020. *PLoS One*. 2022;17(3 March):e0265434. doi:10.1371/journal.pone.0265434

136. Rao A, Lesko C, Mhlophe H, et al. Longitudinal patterns of initiation, persistence, and cycling on preexposure prophylaxis among female sex workers and adolescent girls and young women in South Africa. *AIDS*. 2023;37(6):977-986. doi:10.1097/QAD.0000000000003500

137. Rao A, Mhlophe H, Pretorius A, et al. Effect of implementation strategies on pre-exposure prophylaxis persistence among female sex workers in South Africa: an interrupted time series study. *Lancet HIV*. 2023;10(12):e807-e815. doi:10.1016/S2352-3018(23)00262-X

138. Ridgway JP, Devlin SA, Friedman EE, et al. POWER Up-improving pre-exposure prophylaxis (PrEP) uptake among Black cisgender women in the southern United States: protocol for a stepped-wedge cluster randomized trial (SW-CRT). *PLoS One*. 2023;18(5 May):e0285858. doi:10.1371/journal.pone.0285858

139. Riley T, Anaya G, Gallegos PA, Castaneda R, Khosropour CM. Pre-exposure Prophylaxis Use and Discontinuation in a Federally Qualified Health Center in a Mexico-US Border City. *J Racial Ethn Health Disparities*. Published online October 3, 2023. doi:10.1007/s40615-023-01807-y

140. Rolle CPM, Onwubiko U, Jo J, Sheth AN, Kelley CF, Holland DP. Prep implementation and persistence in a county health department in Atlanta, GA. *Top Antivir Med*. 2018;26:461s. https://www.embase.com/search/results?subaction=viewrecord&id=L621728885&from=export

141. Rousseau E, Wu L, Heffron R, et al. Association of sexual relationship power with PrEP persistence and other sexual health outcomes among adolescent and young women in Kenya and South Africa. *Front Reprod Health*. 2023;5:1073103. doi:10.3389/frph.2023.1073103

142. Rotsaert A, Essack Z, Bosman S, Davey DJ, Hensen B. Oral pre-exposure prophylaxis initiation, continuation and adherence among pregnant and postpartum women receiving antenatal and postnatal care: a systematic review. *J Int AIDS Soc*. 2025;28 Suppl 5(S5):e70035. doi:10.1002/jia2.70035

143. Rugira E, Biracyaza E, Umubyeyi A. Uptake and Persistence on HIV Pre-Exposure Prophylaxis Among Female Sex Workers and Men Having Sex with Men in Kigali, Rwanda: A Retrospective Cross-Sectional Study Design. Patient Prefer Adherence. 2023;17:2353-2364. doi:10.2147/PPA.S427021

144. Rutstein SE, Matoga M, Chen JS, et al. Integrating Enhanced HIV Pre-exposure Prophylaxis Into a Sexually Transmitted Infection Clinic in Lilongwe: Protocol for a Prospective Cohort Study. *JMIR Res Protoc*. 2022;11(12):e37395. doi:10.2196/37395

145. Rutstein SE, Chen JS, Jere E, et al. High incidence of curable sexually transmitted infections among persons accessing oral preexposure prophylaxis at an integrated sexually transmitted infection/preexposure prophylaxis clinic in Lilongwe, Malawi. *Sex Transm Dis*. 2025;52(9):577-584. doi:10.1097/OLQ.0000000000002170

146. Sevelius J, Veras MASM, Gomez JL, et al. Reducing intersectional stigma among transgender women in Brazil to promote uptake of HIV testing and PrEP: study protocol for a randomised controlled trial of Manas por Manas. *BMJ Open*. 2024;14(6):e076878. doi:10.1136/bmjopen-2023-076878

147. Shaikh S, Mugundu Ramien P, Bell J, et al. Laser hair removal to antiretrovirals: findings from a person-centred care model for transgender people in India. *J Int AIDS Soc*. 2025;28 Suppl 5(S5):e70041. doi:10.1002/jia2.70041

148. Shangase N, Jiyane A, Buthelezi F, O’Connor C, Brown B, Rees K. Pre-exposure prophylaxis persistence at two sites in an integrated primary health care programme in South Africa. *Front Public Health*. 2025;13:1460180. doi:10.3389/fpubh.2025.1460180

149. Sharpe JD, Siegler AJ, Sanchez TH, Guest JL, Sullivan PS. Effects of mode of transportation on PrEP persistence among urban men who have sex with men. *AIDS Care*. 2023;35(9):1411-1419. doi:10.1080/09540121.2023.2217375

150. Spinelli MA, Scott HM, Vittinghoff E, et al. Missed Visits Associated with Future Preexposure Prophylaxis (PrEP) Discontinuation among PrEP Users in a Municipal Primary Care Health Network. *Open Forum Infect Dis*. 2019;6(4):ofz101. doi:10.1093/ofid/ofz101

151. Spinelli MA, Buchbinder SP. Pre-exposure prophylaxis persistence is a critical issue in prep implementation. *Clin Infect Dis*. 2020;71(3):583-585. doi:10.1093/cid/ciz896

152. Spinelli MA, Laborde N, Kinley P, et al. Missed opportunities to prevent HIV infections among pre-exposure prophylaxis users: a population-based mixed methods study, San Francisco, United States. *J Int AIDS Soc*. 2020;23(4):e25472. doi:10.1002/jia2.25472

153. Spinelli MA, Laborde N, Kinley P, et al. The importance of PrEP persistence in preventing HIV infections on PrEP. *J Int AIDS Soc*. 2020;23(8):e25578. doi:10.1002/jia2.25578

154. Storholm ED, Ogunbajo A, Nacht CL, et al. Facilitators of PrEP Persistence among Black and Latinx Transgender Women in a PrEP Demonstration Project in Southern California. *Behav Med*. 2022;50(1):63-74. doi:10.1080/08964289.2022.2105794

155. Storholm ED, Siconolfi DE, Wagner GJ, et al. Intimate Partner Violence and HIV Prevention Among Sexual Minority Men: Protocol for a Prospective Mixed Methods Cohort Study. *JMIR Res Protoc*. 2022;11(11):e41453. doi:10.2196/41453

156. Storholm ED, Huang W, Ogunbajo A, et al. Gender-Based Violence and Post-traumatic Stress Disorder Symptoms Predict HIV PrEP Uptake and Persistence Failure Among Transgender and Non-binary Persons Participating in a PrEP Demonstration Project in Southern California. *AIDS Behav*. 2022;27(2):745-759. doi:10.1007/s10461-022-03807-1

157. Sullivan PS, Hall E, Bradley H, Russell ES, Woodyatt CR. Inequities in PrEP annualized pill-day coverage, United States, 2018-2022: a cross-sectional pharmacoequity analysis. *J Int AIDS Soc*. 2025;28(5):e26459. doi:10.1002/jia2.26459

158. Sutten Coats C, Goedel WC, Sims-Gomillia CE, et al. “Make it more than a pill, make it an experience of health:” results from an open pilot intervention to retain young African American men who have sex with men in PrEP care. *AIDS Care*. 2024;36(4):472-481. doi:10.1080/09540121.2023.2221422

159. Tanner MR, Miele P, Carter W, et al. Preexposure prophylaxis for prevention of HIV acquisition among adolescents: Clinical considerations, 2020. *MMWR Recomm Rep*. 2020;69(3):1-12. doi:10.15585/MMWR.RR6903A1

160. Tao J, Gu M, Galarraga O, et al. Long-term HIV pre-exposure prophylaxis persistence and reinitiation in Connecticut from 2012 to 2018. *Popul Health Manag*. 2024;27(4):267-274. doi:10.1089/pop.2024.0012

161. Tapsoba J de D, Zangeneh SZ, Appelmans E, et al. Persistence of oral pre-exposure prophylaxis (PrEP) among adolescent girls and young women initiating PrEP for HIV prevention in Kenya. *AIDS Care*. 2020;33(6):712-720. doi:10.1080/09540121.2020.1822505

162. Vanbaelen T, Rotsaert A, Jacobs BKM, et al. Why do HIV pre-exposure prophylaxis users discontinue pre-exposure prophylaxis care? A mixed methods survey in a pre-exposure prophylaxis clinic in Belgium. *AIDS Patient Care STDS*. 2022;36(4):159-167. doi:10.1089/apc.2021.0197

163. Vitruk O, Ihnatiuk AP, Kazanzhy AP, et al. Uptake and persistent use of HIV preexposure prophylaxis among key populations: Results from Ukraine’s scaled national preexposure prophylaxis program. *J Acquir Immune Defic Syndr*. 2024;96(3):241-249. doi:10.1097/QAI.0000000000003432

164. Vu BN, Green K, Phan H, et al. Engaging the private sector as part of HIV pre-exposure prophylaxis service delivery in Vietnam: a comparative analysis of uptake, persistence and HIV seroconversion from 2018 to 2023. *Front Reprod Health*. 2024;6:1439461. doi:10.3389/frph.2024.1439461

165. Waetjen M, Papadopoulou M, Flores R, et al. Pre-exposure Prophylaxis Persistence Among Greek Sexual Minority Men: Results from PrEP for Greece (P4G) Study. *AIDS Behav*. 2021;26(4):1039-1046. doi:10.1007/s10461-021-03459-7

166. Weir BW, Wirtz AL, Chemnasiri T, et al. High PrEP uptake, adherence, persistence and effectiveness outcomes among young Thai men and transgender women who sell sex in Bangkok and Pattaya, Thailand: findings from the open-label combination HIV prevention effectiveness (COPE) study. *Lancet Reg Health Southeast Asia*. 2023;15:100217. doi:10.1016/j.lansea.2023.100217

167. Wheatley MM, Knowlton G, Kao SY, Jenness SM, Enns EA. Cost-effectiveness of interventions to improve HIV pre-exposure prophylaxis initiation, adherence, and persistence among men who have sex with men. *J Acquir Immune Defic Syndr Hum Retrovirol*. 2022;90(1):41-49. doi:10.1097/QAI.0000000000002921

168. Whelchel K, Zuckerman AD, DeClercq J, Choi L, Kelly SG. Optimizing HIV PrEP Persistence: Does Your Pharmacy Matter? *AIDS Behav*. 2023;27(11):3735-3744. doi:10.1007/s10461-023-04091-3

169. Wray TB, Chan PA, Kahler CW, Ocean EMS, Nittas V. Pilot Randomized Controlled Trial of Game Plan for PrEP: A Brief, Web and Text Message Intervention to Help Sexual Minority Men Adhere to PrEP and Reduce Their Alcohol Use. *AIDS Behav*. 2023;28(4):1356-1369. doi:10.1007/s10461-023-04223-9
